# Electron transport chain inhibition increases cellular dependence on purine transport and salvage

**DOI:** 10.1016/j.cmet.2024.05.014

**Published:** 2024-06-13

**Authors:** Zheng Wu, Divya Bezwada, Feng Cai, Robert C Harris, Bookyung Ko, Varun Sondhi, Chunxiao Pan, Hieu S. Vu, Phong T Nguyen, Brandon Faubert, Ling Cai, Hongli Chen, Misty Martin-Sandoval, Duyen Do, Wen Gu, Yuanyuan Zhang, Yuannyu Zhang, Bailey Brooks, Sherwin Kelekar, Lauren G Zacharias, K. Celeste Oaxaca, Joao S Patricio, Thomas P Mathews, Javier Garcia-Bermudez, Min Ni, Ralph J. DeBerardinis

**Affiliations:** 1 Children’s Medical Center Research Institute, University of Texas Southwestern Medical Center, Dallas, TX, 75390, USA; 2 Division of Pulmonary and Critical Care Medicine, Department of Internal Medicine, UT Southwestern Medical Center, Dallas, TX, 75390, USA; 3 Quantitative Biomedical Research Center, Department of Population and Data Sciences, UT Southwestern Medical Center, Dallas, TX, 75390, USA; 4 Simmons Comprehensive Cancer Center, UT Southwestern Medical Center, Dallas, TX, 75390, USA; 5 Department of Medicine, University of Chicago, Chicago, IL, 60637, USA; 6 Department of Radiation Oncology, UT Southwestern Medical Center, Dallas, 75390, TX, USA; 7 Harold C. Simmons Comprehensive Cancer Center, UT Southwestern Medical Center, Dallas, TX, 75390, USA; 8 Howard Hughes Medical Institute, University of Texas Southwestern Medical Center, Dallas, TX, 75390, USA; 9 Lead contact

## Abstract

Mitochondria house many metabolic pathways required for homeostasis and growth. To explore how human cells respond to mitochondrial dysfunction, we performed metabolomics in fibroblasts from patients with various mitochondrial disorders and cancer cells with electron transport chain (ETC) blocakde. These analyses revealed extensive perturbations in purine metabolism, and stable isotope tracing demonstrated that ETC defects suppress de novo purine synthesis while enhancing purine salvage. In human lung cancer, tumors with markers of low oxidative mitochondrial metabolism exhibit enhanced expression of the salvage enzyme hypoxanthine phosphoribosyl transferase 1 (HPRT1) and high levels of the HPRT1 product inosine monophosphate. Mechanistically, ETC blockade activates the pentose phosphate pathway, providing phosphoribosyl diphosphate to drive purine salvage supplied by uptake of extracellular bases. Blocking HPRT1 sensitizes cancer cells to ETC inhibition. These findings demonstrate how cells remodel purine metabolism upon ETC blockade and uncover a new metabolic vulnerability in tumors with low respiration.

## eTOC

Wu et al. report that mitochondrial electron transport chain impairment induces a metabolic shift from de novo purine biosynthesis to purine salvage. Cancer cells with low electron transport chain activity require purine uptake and salvage to grow in culture and in vivo.

## Graphical Abstract

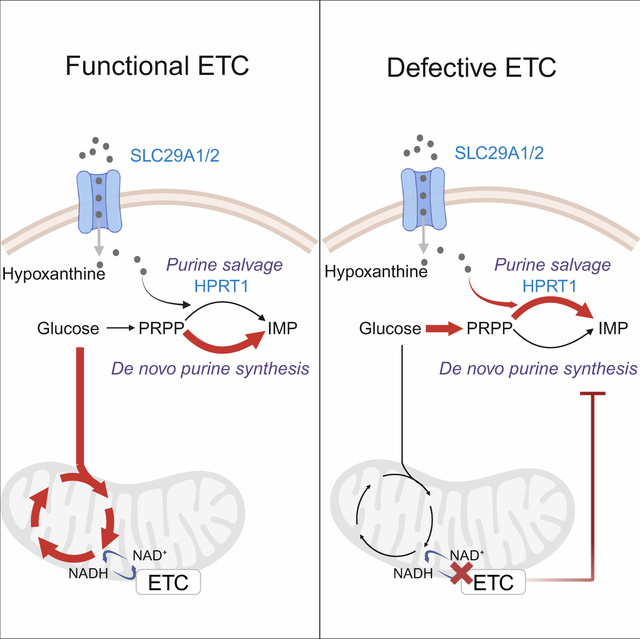

## INTRODUCTION

Cancer and inborn errors of metabolism (IEMs) are characterized by mutations that perturb cellular metabolism. Although cancer and IEMs are clinically very different, they share some pathogenic mechanisms. Oncogenic signaling regulates many of the same metabolic pathways that become dysfunctional in IEMs, including glycolysis, amino acid oxidation, the urea cycle and others^1–4^. Dozens of human IEMs are caused by mutations of mitochondrial enzymes, particularly in the TCA cycle and subunits of the electron transport chain (ETC) necessary for oxidative phosphorylation (OXPHOS)^5^. Some of these same enzymes, including succinate dehydrogenase and fumarate hydratase, are tumor suppressors in adult-onset cancers^6–8^. Therefore, studying metabolic reprogramming in cancer can provide insights relevant to the pathophysiology of IEMs, and vice versa^9^.

Mitochondria house pathways that contribute to cell growth by producing energy, biosynthetic precursors, and signaling molecules. Human tumors appear to vary in their need for OXPHOS, with some showing genetic evidence for suppression of ETC function^6,8,10–12^. However, although many tumors contain nonsynonymous mitochondrial DNA point mutations, including ones predicted to impair the ETC, tumors often select against these mutations as they progress^13,14^. Efforts to assess human lung tumor metabolism in vivo using intra-operative ^13^C-glucose infusions demonstrate variable ^13^C labeling of TCA cycle intermediates among tumors from different patients and in different regions of the same tumor^15,16^. This variation could be related to intrinsic properties of tumor cells^17,18^, or to environmental factors such as hypoxia, which may limit oxidation of glucose and other nutrients. In mice, tumors display low tricarboxylic acid (TCA) cycle turnover at the site of origin, but higher activity at some metastatic sites^19^. Genetically eliminating ETC components severely suppresses xenograft growth, even at the site of implantation, indicating the requirement for at least some OXPHOS in those models^20,21^.

A marker of ETC dysfunction relevant to cancer cell growth is NADH reductive stress (i.e., low NAD^+^:NADH ratio), which occurs when NADH production outpaces its oxidation to NAD^+^, particularly by ETC Complex I. This form of metabolic stress has broad implications for intermediary metabolism because many NAD(H)-dependent oxidoreductases are sensitive to the NAD^+^:NADH ratio. High demands for NAD^+^ can impose a bottleneck on growth because some pathways that produce precursors for macromolecular synthesis contain NAD^+^-dependent oxidoreductases^22,23^. The Warburg effect, which describes the conversion of glucose to lactate in the presence of oxygen observed in most cancer cells, is thought to reflect the need to regenerate NAD^+^ by lactate dehydrogenase when mitochondrial redox shuttles become oversaturated^24^. Identifying consistent responses to low redox ratios may provide new insights about metabolic diseases, including cancer.

Here we sought to identify consistent metabolic alterations downstream of mitochondrial dysfunction in humans. We report that ETC dysfunction changes how cells produce purines, with the salvage enzyme HPRT1 becoming essential for growth in the setting of mitochondrial impairment.

## RESULTS

### Altered purine metabolism is a common feature of human mitochondrial disease

**Figure 1.**
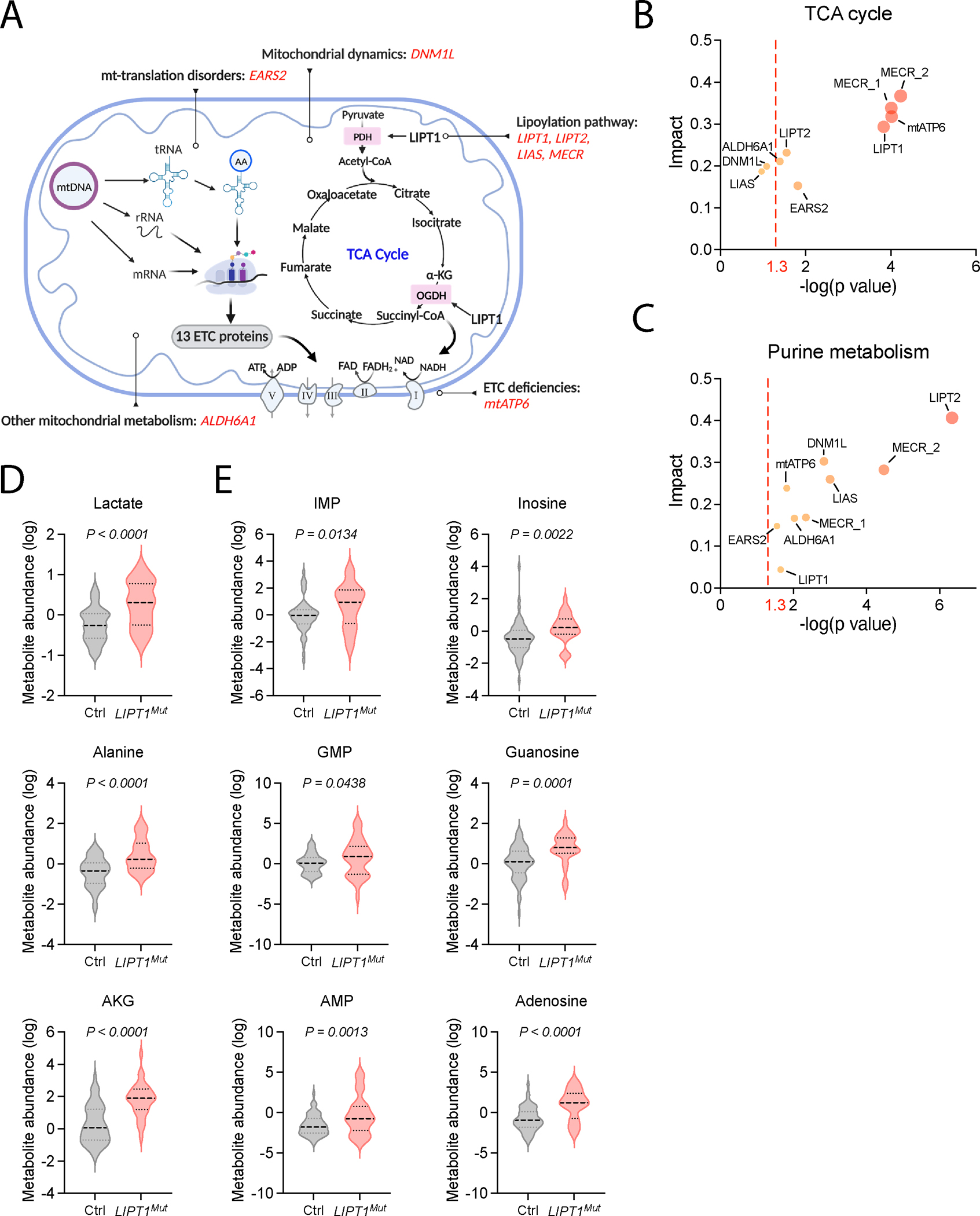
Metabolomic profiling of patients with mitochondrial disorders. **A.** Illustration of the human mitochondrial defects analyzed in panels B-E. **B.** and **C.** Altered metabolite abundances in the TCA cycle (**B**) and purine metabolism (**C**) in fibroblasts from patients with the indicated mitochondrial defects. Each dot represents a fibroblast line from a patient with a disorder affecting the mitochondria. The mutated gene from each disorder is indicated. **D. and E.** Plasma metabolite levels from a patient with LIPT1 deficiency and healthy controls. n = 60 (healthy); n = 28 (LIPT1 deficiency; samples collected on different days). An unpaired, two-sided t test was used for the statistical analysis. BioRender was used to generate the illustration.

To identify the effects of mitochondrial dysfunction in humans, we first analyzed metabolomics in fibroblasts from patients with genetic diseases affecting mitochondrial RNA processing and translation, lipoylation of mitochondrial enzymes, the ETC or other processes (Figure 1A). Metabolite set enrichment analysis (MSEA) revealed that the TCA cycle is commonly perturbed in fibroblasts from these patients (Figure 1B). Unexpectedly, even more cells from this panel display alterations in purine metabolism (Figure 1C). We also analyzed plasma from a previously-reported patient with Lipoyltransferase-1 (LIPT1) deficiency, which results in dysfunction of pyruvate dehydrogenase and oxoglutarate dehydrogenase (PDH and OGDH)^25^. We compared plasma collected from this patient during several hospitalizations to plasma from healthy controls. As expected, plasma from the LIPT1-deficient patient had elevated lactate, alanine, and α-ketoglutarate (Figure 1D). Several purine metabolites were also increased in plasma from this patient (Figure 1E).

### ETC blockade increases purine metabolites

**Figure 2.**
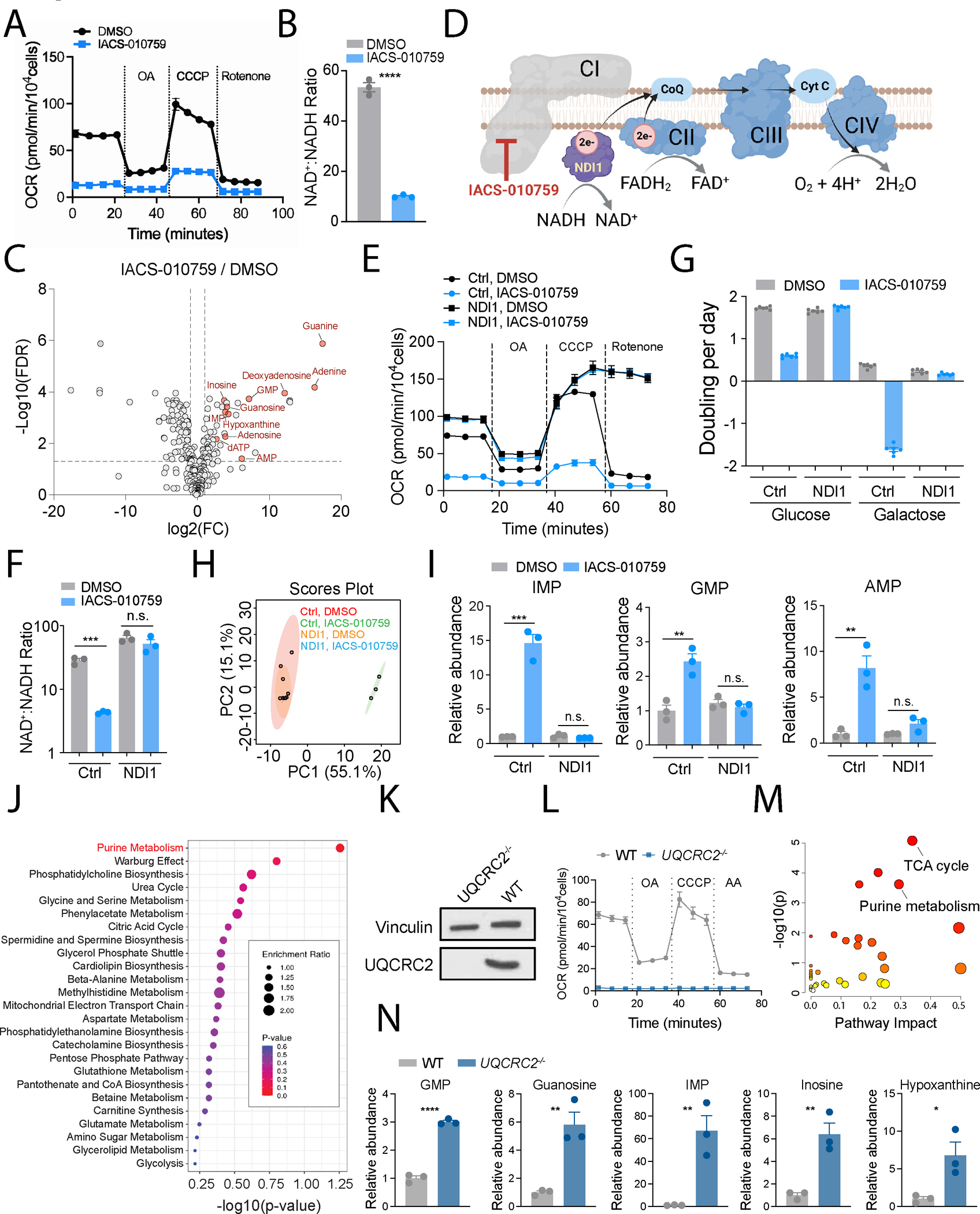
Mitochondrial ETC deficiency causes accumulation of purine metabolites. **A.** Oxygen consumption rates (OCR) of H460 cells pre-treated with DMSO or 25 nM IACS-010759. OA: oligomycin A; CCCP: Carbonyl cyanide *m*-chlorophenylhydrazone. Data represent one of three independent experiments. **B.** NAD^+^:NADH ratio in H460 cells treated with DMSO or 25 nM IACS-010759 for 24 hours (n=3). **C.** Volcano plot showing metabolomic changes in H460 cells treated with DMSO or 25 nM IACS-010759 for 24 hours. The pink circles are increased purine metabolites with FDR < 0.05. **D.** Schematic illustrating the mechanism of NDI1 rescue of ETC complex I blockade. **E.** OCR of control and NDI1-expressing H460 cells pre-treated with DMSO or 25 nM IACS-010759. Data represent one of three independent experiments. **F.** NAD^+^:NADH ratio in control and NDI1-expressing H460 cells treated with DMSO or 25 nM IACS-010759 for 24 hours (n=3). **G.** Growth rates of control and NDI1-expressing cells cultured in glucose or galactose medium and treated with DMSO or 25 nM IACS-010759 (n=6). Data are from one of three independent experiments. **H.** Principal component analysis of metabolomic profiles in control and NDI1-expressing H460 cells treated with DMSO or 25 nM IACS-010759 for 24 hours. **I.** Relative abundance of the indicated purine nucleotides in control and NDI1-expressing H460 cells treated with DMSO or 25 nM IACS-010759 for 24 hours (n=3). **J.** Metabolite set enrichment analysis comparing IACS-010759-treated control and NDI-expressing H460 cells. **K.** Western blot validating deletion of UQCRC2. Vinculin is the loading control. **L.** OCR in WT and *UQCRC2*^*−/−*^ H460 cells. AA; antimycin A. Data are from one of three independent experiments. **M.** Metabolic pathway analysis of differentially abundant metabolites in *UQCRC2*-depleted (*UQCRC2*^*−/−*^) H460 cells compared to parental cells. **N.** Relative abundance of the indicated purine metabolites in WT and *UQCRC2*^*−/−*^ H460 cells (n=3). Unpaired, two-sided t tests were used for the statistical analyses. ****: P < 0.0001; ***: P < 0.001, **: P < 0.01, *: P < 0.05; n.s.: P > 0.05. Error bars denote SEM. BioRender was used to generate the illustration.

To assess mitochondrial dysfunction in a simpler system, we treated human H460 non-small cell lung cancer (NSCLC) cells with IACS-010759, a potent mitochondrial ETC complex I inhibitor (Figure 2A)^26^. The drug reduced the cellular NAD^+^:NADH ratio (Figure 2B) and induced a compensatory increase in glucose uptake and lactate secretion (Figures S1A and S1B). Metabolomics revealed widespread effects of IACS-010759, including reduced levels of several metabolites related to TCA cycle function (Figure S1C and S1D)^27–29^. Many purine metabolites were elevated upon IACS-010759 treatment, despite the depletion of aspartate, a precursor for de novo purine synthesis (Figures 2C and S1D). IACS-010759 also increased purine monophosphates in other cell lines (Figures S1E and S1F). To test whether IACS-010759 impacts purine metabolism in NSCLC xenografts in vivo, H460 cells were subcutaneously implanted into immunocompromised mice, and metabolomics was performed after treating the mice with IACS-010759 or vehicle. Among many alterations, purine metabolism stood out as by far the most affected pathway, with marked accumulations in GMP and guanosine (Figure S1G–S1I).

To verify that IACS-010759 induced these changes through complex I inhibition, we expressed *Saccharomyces cerevisiae* alternative NADH dehydrogenase (NDI1) to restore OXPHOS (Figure 2D). NDI1 boosted basal mitochondrial respiration in H460 cells, rendered them resistant to IACS-010759 and rotenone, and normalized the NAD^+^:NADH ratio, glucose uptake, and lactate secretion (Figure 2E, F, S2A and S2B). Control H460 cells expressing an empty vector displayed growth suppression upon IACS-010759 treatment, and extensive cell death in medium containing galactose instead of glucose. These effects were reversed by NDI1 (Figure 2G). Metabolomics revealed that nearly all IACS-010759-induced metabolic alterations, including those involving purines, were corrected by NDI1 (Figures 2H, 2I and S2C). MSEA identified purine metabolism as the top-scoring pathway from metabolites that were altered by IACS-010759 in control but not NDI1-expressing cells (Figure 2J), identifying this pathway as the most responsive to complex I blockade.

To evaluate whether altered purine metabolism is a common phenotype associated with defects in other ETC complexes, we generated H460 cell lines depleted for UQCRC2, a component of ETC complex III (i.e., *UQCRC2*^*−/−*^ cells, Figure 2K). UQCRC2 ablation impaired mitochondrial respiration (Figure 2L), suppressed cell growth (Figure S2D), and induced a distinct metabolomic profile (Figures S2E and S2F), including marked purine accumulation (Figures 2M and 2N).

Reduced cell proliferation in cells with ETC defects could result in suppressed purine consumption for nucleic acid synthesis, thereby leading to purine accumulation. To examine the relationship between proliferation and purine accumulation, we treated H460 cells with nocodazole, an inhibitor of microtubule polymerization, at a dose that arrests cell proliferation (Figure S2G). This had no effect on purine monophosphate levels (Figure S2H). We then used SK-N-DZ neuroblastoma cells, which maintain proliferation despite reduced oxygen consumption during IACS-010759 treatment^30^ (Figures S2I and S2J). These cells still accumulated purine monophosphates when complex I was inhibited (Figure S2K). Therefore, although ETC blockade can suppress growth, this is neither sufficient nor necessary to induce purine nucleotide accumulation.

### Cytosolic NAD^+^:NADH ratio impacts purine metabolism upon ETC blockade

**Figure 3.**
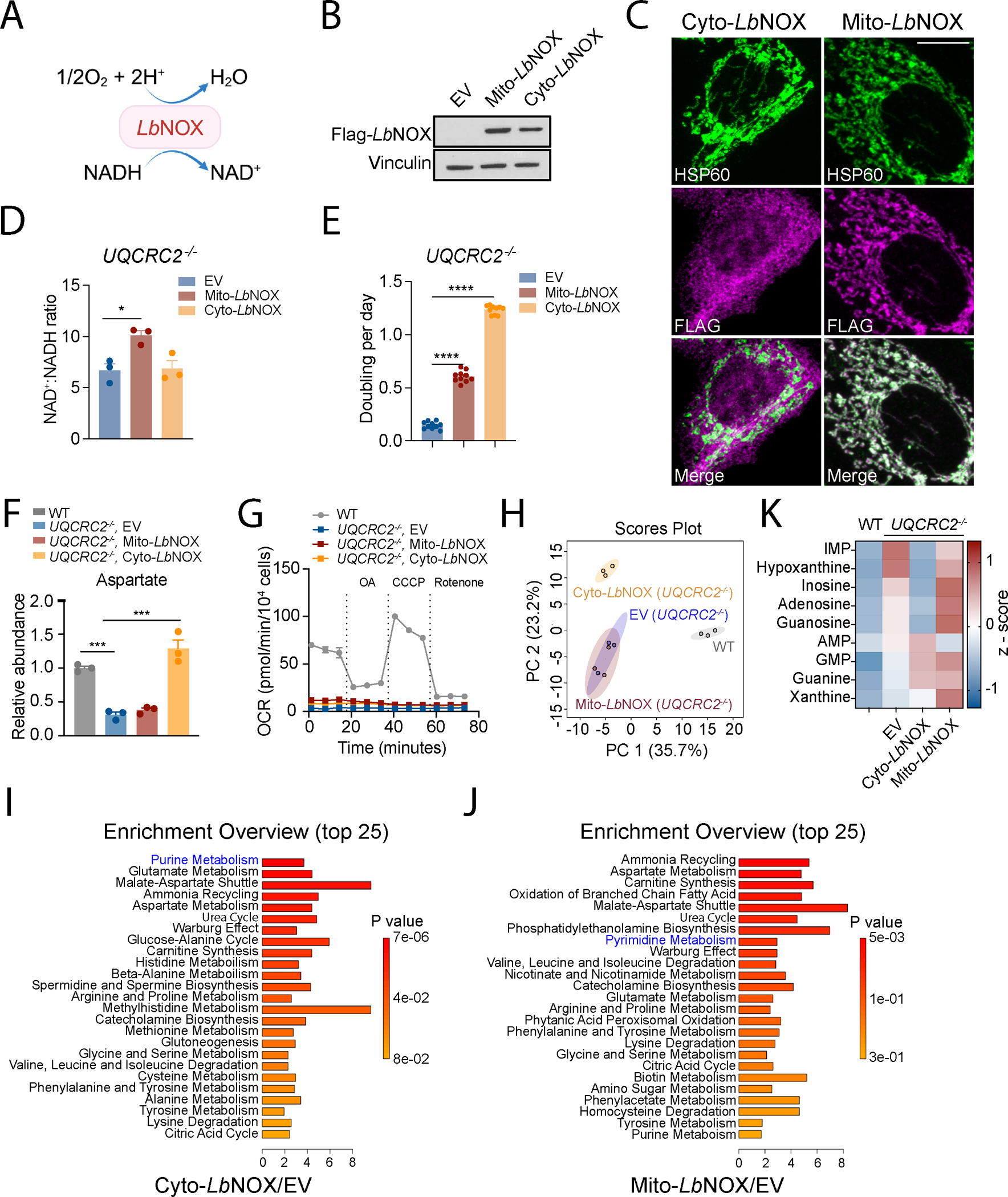
Cytosolic NAD(H) imbalance impacts purine accumulation in ETC-deficient cells. **A.** Schematic of *Lb*NOX-catalyzed reaction. **B.** Western blot validating expression of Flag-tagged *Lb*NOX in *UQCRC2*^*−/−*^ H460 cells. Vinculin is the loading control. **C.** Immunofluorescence showing the subcellular localization of the indicated Flag-tagged *Lb*NOX proteins in *UQCRC2*^*−/−*^ H460 cells. HSP60 is a mitochondrial matrix marker. Scale bar represents 10 μm. **D.** NAD^+^:NADH ratio in *UQCRC2*^*−/−*^ cells expressing empty vector (EV), Mito-*Lb*NOX or Cyto-*Lb*NOX (n=3). **E.** Growth rates of *UQCRC2*^*−/−*^ H460 cells expressing empty vector (EV), Mito-*Lb*NOX, or Cyto-*Lb*NOX (n=10). Data are from one of three independent experiments. **F.** Relative abundance of aspartate in WT H460 cells and *UQCRC2*^*−/−*^ H460 cells expressing empty vector (EV), Mito-*Lb*NOX or Cyto-*Lb*NOX (n=3). **G.** OCR of WT and *UQCRC2*^*−/−*^ cells expressing empty vector (EV), Mito*-Lb*NOX or Cyto*-Lb*NOX. Data are from one of three independent experiments. **H.** Principal component analysis of metabolomic profiles in WT and *UQCRC2*^*−/−*^ cells expressing empty vector (EV), Mito*-Lb*NOX, or Cyto*-Lb*NOX. **I.** Metabolite set enrichment analysis comparing *UQCRC2*^*−/−*^ cells expressing empty vector (EV) or Cyto*-Lb*NOX. **J.** Metabolite set enrichment analysis comparing *UQCRC2*^*−/−*^ cells expressing empty vector (EV) or Mito*-Lb*NOX. **K.** Heatmap displaying metabolite abundance in WT H460 cells and *UQCRC2*^*−/−*^ H460 cells expressing empty vector (EV), Mito-*Lb*NOX or Cyto-*Lb*NOX. Unpaired, two-sided t tests were used for the statistical analyses. ****: P < 0.0001, ***: P < 0.001, **: P < 0.01, *: P < 0.05. Error bars denote SEM. BioRender was used to generate the illustration.

Mitochondrial respiration is coupled to oxidation of reducing equivalents (i.e., NADH and FADH_2_), ATP synthesis, and mitochondrial membrane potential. It was unclear which of these modulates purine abundance. Given that NAD^+^ is required for many oxidoreductase reactions, we reasoned that the decreased NAD^+^:NADH ratio impacts purine metabolism when the ETC is compromised. To dissociate NADH oxidation from OXPHOS, we expressed the water-forming NADH oxidase from *Lactobacillus brevis* (*Lb*NOX) that utilizes oxygen to convert NADH to NAD^+^ (Figure 3A)^31^. We localized *Lb*NOX to cytosol or mitochondria in *UQCRC2*^*−/−*^ cells to ameliorate NADH accumulation in either compartment (Figures 3B and 3C)^31^. In whole-cell lysates, Mito-*Lb*NOX but not Cyto-*Lb*NOX increased the NAD^+^:NADH ratio (Figure 3D). Nevertheless, consistent with previous studies^31^, both versions improved *UQCRC2*^*−/−*^ cell growth in the absence of pyruvate and uridine, and this was more pronounced with Cyto-*Lb*NOX (Figure 3E). Cyto-*Lb*NOX also enhanced aspartate abundance to a greater extent (Figure 3F). Importantly, *Lb*NOX did not restore oxygen consumption in *UQCRC2*^*−/−*^ cells, indicating that it increases the NAD^+^:NADH ratio independently of OXPHOS (Figure 3G). We assessed metabolomic profiles of WT and *UQCRC2*^*−/−*^ cells expressing either an empty vector (EV) or Mito/Cyto-*Lb*NOX, all grown without pyruvate and uridine supplementation. Under these conditions, Cyto-*Lb*NOX had a greater overall metabolomic impact than Mito-*Lb*NOX (Figure 3H), although Mito-*Lb*NOX had a more pronounced effect on glutamine reductive carboxylation (Figure S3A). Neither Cyto-*Lb*NOX nor Mito-*Lb*NOX completely alleviated the metabolomic effects of UQCRC2 loss (Figure S3B). MSEA identified numerous pathways modulated by Mito-*Lb*NOX and Cyto-*Lb*NOX in ETC-deficient cells (Figure 3I and 3J). However, in terms of nucleotide metabolism, Cyto-*Lb*NOX primarily affected purines while Mito-*Lb*NOX primarily affected pyrimidines (Figure 3I and 3J). Accordingly, Cyto-*Lb*NOX, but not Mito-*Lb*NOX reduced IMP and hypoxanthine levels in *UQCRC2*^*−/−*^ cells (Figure 3K). AMP and GMP were not normalized by Cyto-*Lb*NOX but instead increased further. The increased GMP level is likely attributed to enhanced activity of inosine monophosphate dehydrogenases 1/2 (IMPDH1/2), which require cytosolic NAD^+^ in the GMP synthesis pathway^28^. The elevation in AMP may result from increased availability of aspartate^28^. We also supplemented the medium with α-ketobutyrate (AKB), a compound utilized to mitigate cytosolic NADH accumulation (Figure S3C)^28^. Similar to Cyto-*Lb*NOX, AKB reduced hypoxanthine and IMP levels and enhanced cell growth (Figures S3D and S3E).

### ETC blockade suppresses de novo purine synthesis

**Figure 4.**
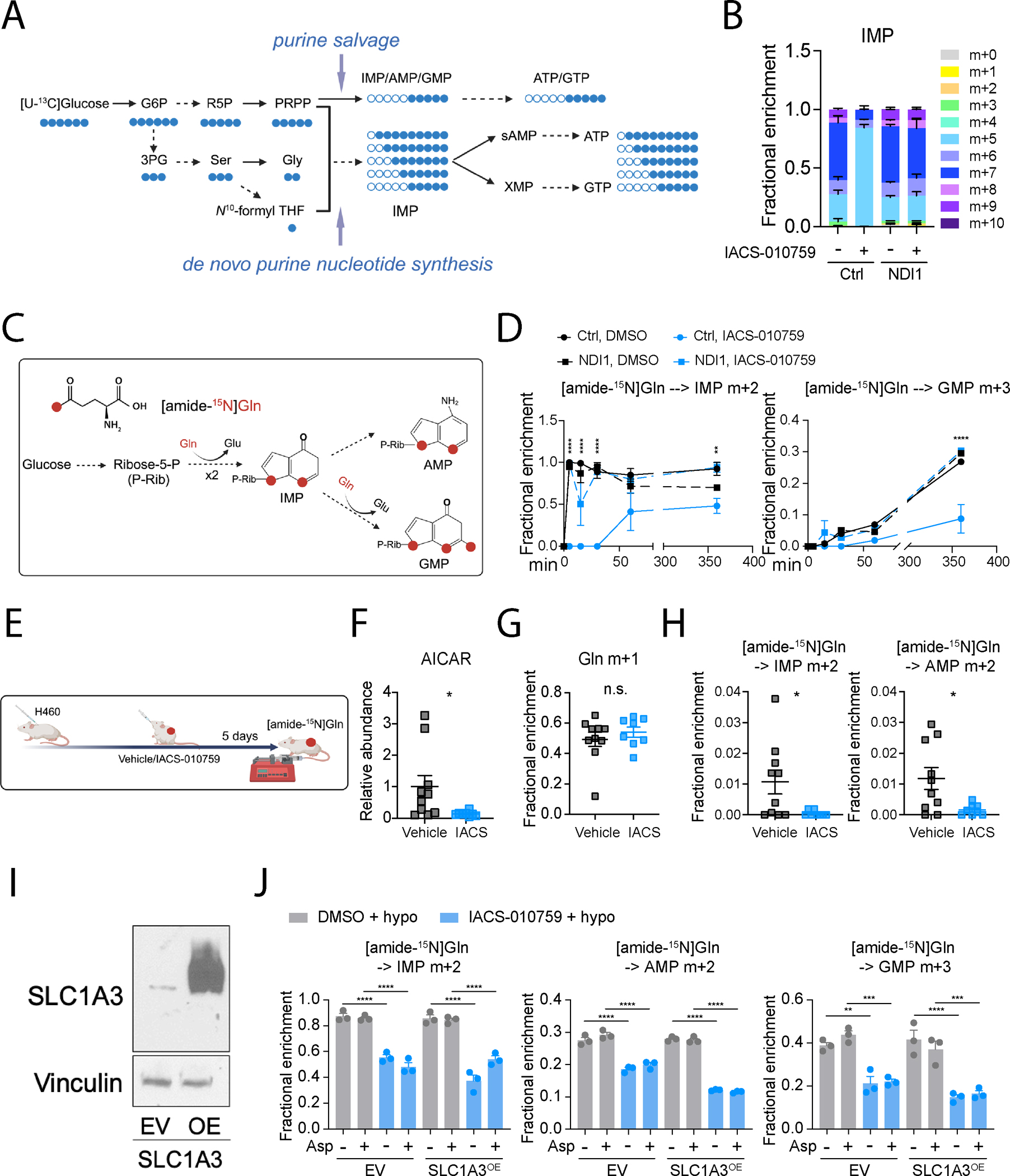
ETC blockade suppresses de novo purine nucleotide synthesis. **A.** Schematic illustrating ^13^C labeling of purines from [U-^13^C]glucose. **B.** ^13^C labeling in IMP after 6 hours of culture with [U-^13^C]glucose in control and NDI1-expressing H460 cells pre-treated with DMSO or 25 nM IACS-010759 for 24 hours (n=3). **C.** Schematic illustrating ^15^N labeling from [amide-^15^N]glutamine during de novo purine nucleotide synthesis. **D.** Time-dependent fractional enrichment of m+2 IMP and m+3 GMP in control and NDI1-expressing H460 cells pre-treated with DMSO or 25 nM IACS-010759 for 24 hours (n =3 at each time point). **E.** Schematic illustrating infusion of [amide-^15^N]glutamine into mice bearing H460 xenografts. **F.** Relative abundance of AICAR in H460 xenografts treated with vehicle or IACS-010759 for 5 days. Vehicle (n=10), IACS (n=8). **G-H.** Fractional enrichment of m+1 glutamine (**G**), m+2 IMP and m+2 AMP (**H**) in vehicle and IACS-010759-treated H460 xenografts after 4 hours of [amide-^15^N]glutamine infusion. Vehicle (n=10), IACS (n=8). **I.** Western blot validating overexpression of SLC1A3. Vinculin is the loading control. EV: empty vector; OE: overexpression. **J.** Fractional enrichment of m+2 IMP, m+2 AMP, and m+3 GMP after 6 hours of culture with [amide-^15^N]glutamine in empty vector-expressing control cells (EV) and SLC1A3-overexpressing (SLC1A3^OE^) cells pretreated with or without DMSO, 25 nM IACS-010759, 150 μM aspartate (Asp), and 10 μM hypoxanthine (hypo) (n=3). Unpaired, two-sided t tests (**F-H**), two-way ANOVA (**D**), and one-way ANOVA (**J**) were used for the statistical analyses. ****: P < 0.0001; ***: P < 0.001, **: P < 0.01; *: P < 0.05; n.s.: P > 0.05. Error bars denote SEM. BioRender was used to generate the illustration.

To further examine purine metabolism in cells with ETC dysfunction, we cultured cells with uniformly ^13^C-labeled glucose ([U-^13^C]glucose). While de novo purine synthesis yields various purine nucleotide isotopologues reflecting labeling in both the ribose backbone and purine base, purines produced from the salvage pathway are dominated by m+5 labeling in the ribose backbone (Figure 4A). Vehicle-treated cells displayed the expected heterogeneity in IMP isotopologues, but labeling was almost entirely m+5 in IACS-010759-treated cells (Figure 4B). NDI1 eliminated the effect of IACS-010759 on purine labeling (Figure 4B). Similar effects occurred in GTP and ATP, although overall labeling was lower than for IMP (Figure S4A). IACS-010759 also increased time-dependent m+5 labeling of both IMP and GMP (Figure S4B).

We next conducted kinetic [amide-^15^N]glutamine tracing in H460 cells with or without IACS-010759 to assess the de novo purine synthesis pathway through which two and three ^15^N nuclei are incorporated into IMP and GMP, respectively (Figure 4C). Compared to untreated cells, control cells treated with IACS-010759 exhibited lower fractional enrichment of m+2 IMP and m+3 GMP throughout the time course, and this was reversed by NDI1 (Figure 4D). Adding hypoxanthine to the medium did not reverse the suppressed labeling caused by IACS-010759 (Figure S4C). IACS-010759 nearly eliminated 5-aminoimidazole-4-carboxamide ribonucleotide (AICAR) and phosphoribosylaminoimidazolesuccinocarboxamide (SAICAR), two intermediates in the de novo purine synthesis pathway (Figure S4D), as previously observed in ETC Complex III-deficient cells^28^. It also suppressed m+2 labeling of AICAR in control but not NDI1-expressing cells (Figure S4E). These findings indicate a suppression of de novo purine synthesis upon IACS-010759 treatment.

As an orthogonal analysis of the relationship between purine metabolism and the ETC, we used the Cancer Dependency Map (DepMap) to identify co-essential genes with *PPAT^32^*, which encodes Phosphoribosyl Pyrophosphate Amidotransferase, the rate limiting enzyme of the de novo purine synthesis pathway. We observed a strong correlation of PPAT with genes involved in purine metabolism and other related pathways, including the pentose phosphate pathway and one carbon metabolism (Figure S4F). Notably, the highest-scoring pathways were the TCA cycle and oxidative phosphorylation, indicating a critical role of mitochondrial oxidative metabolism in supporting de novo purine synthesis (Figure S4F). IACS-010759 had little effect on mRNA or protein abundance of enzymes in the de novo purine synthesis pathway (Figure S4G and S4H), as expected if pathway suppression occurred as a metabolic effect of ETC inhibition.

To test whether ETC blockade impacts purine metabolism in tumors in vivo, H460 xenograft-bearing mice were dosed with IACS-010759 and infused with [amide-^15^N]glutamine (Figure 4E). AICAR abundance declined in tumors treated with IACS-010759 (Figure 4F). IACS-010759 had no effect on tumor enrichment of m+1 glutamine (Figure 4G) but it suppressed m+2 labeling in IMP and AMP (Figure 4H), indicating suppressed de novo purine synthesis in vivo.

Defective mitochondrial respiration limits synthesis of aspartate, which is required for de novo purine synthesis^27,28,33,34^. This raises the possibility that aspartate depletion explains reduced de novo purine synthesis in IACS-010759-treated cells. Previous studies demonstrated that ETC inhibition leads to increased incorporation of exogenous aspartate into nucleotides^34^, but it is unknown whether aspartate supplementation restores de novo purine synthesis in ETC-deficient cells. To test this, we generated isogenic cell lines that overexpress the aspartate transporter SLC1A3 (SLC1A3^OE^) (Figure 4I). These cells display enhanced aspartate uptake from the medium (Figure S4I). However, despite restoration of intracellular aspartate abundance in SLC1A3^OE^ cells exposed to IACS-010759 (Figure S4J), purine nucleotide labeling from [amide-^15^N]glutamine remained low (Figure 4J). Therefore, the ETC supports de novo purine nucleotide synthesis through mechanisms beyond supplying cellular aspartate.

### Mitochondrial ETC deficiency enhances purine salvage

**Figure 5.**
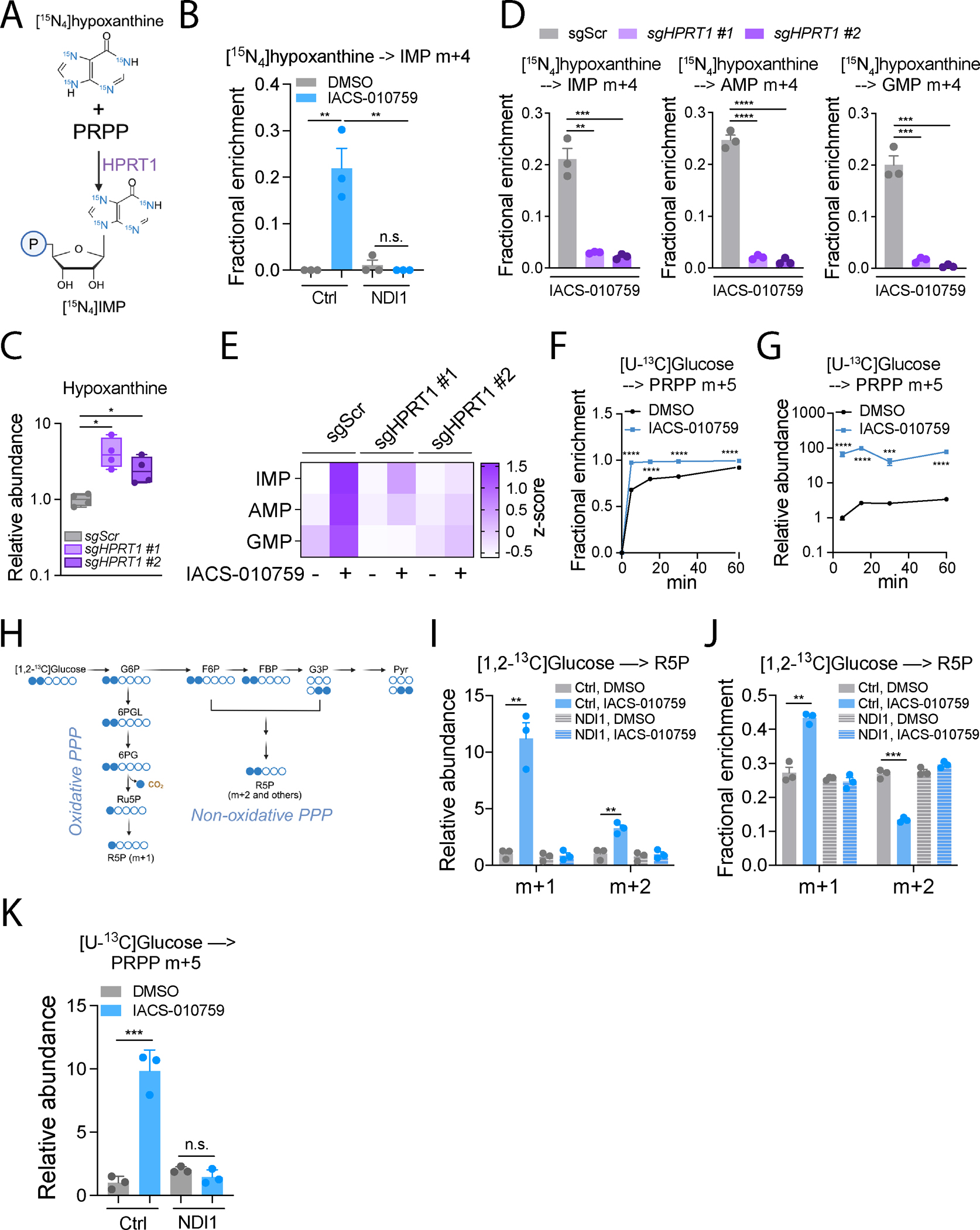
ETC blockade promotes purine salvage. **A.** Schematic illustrating conversion of [^15^N_4_]hypoxanthine to m+4 IMP during HPRT1-mediated salvage. **B.** Fractional enrichment of m+4 IMP from [^15^N_4_]hypoxanthine during 6 hours of tracing in control and NDI1-expressing H460 cells pre-treated with DMSO or 25 nM IACS-010759 for 24 hours. **C.** Relative abundance of hypoxanthine in control (*sgScr*) or HPRT1-depleted (*sgHPRT1*) cells (n=3). **D.** Fractional enrichment of m+4 IMP, AMP, and GMP in control (*sgScr*) or HPRT1-depleted (*sgHPRT1*) cells after 24 hours of pre-treatment with 25 nM IACS-010759 followed by 6 hours of culture with [^15^N_4_]hypoxanthine (n=3). **E.** Heatmap displaying purine nucleotide abundance in control (*sgScr*) or HPRT1-depleted (*sgHPRT1*) cells treated with DMSO or 25 nM IACS-010759 for 24 hours. **F-G.** Time-dependent fractional enrichment (**F**) and relative abundance (**G**) of m+5 PRPP in H460 cells pretreated with DMSO or 25 nM IACS-010759 for 24 hours (n=3 at each time point). **H.** Schematic illustrating ^13^C labeling of R5P from [1,2-^13^C]glucose. **I-J.** Relative abundance (**I**) and fractional enrichment (**J**) of m+1 and m+2 R5P after 6 hours of culture in [1,2-^13^C]glucose. Control and NDI1-expressing H460 cells were pre-treated with DMSO or IACS-010759 for 24 hours (n=3). **K.** Relative abundance of m+5 PRPP after 6 hours of culture in [U-^13^C]glucose. Control and NDI1-expressing H460 cells were pre-treated with DMSO or IACS-010759 for 24 hours (n=3). Unpaired, two-sided t tests (**B**, **D** and **K**), multiple t test (**I** and **J**), and two-way ANOVA (**F** and **G**) were used for the statistical analyses. ****: P < 0.0001; ***: P < 0.001; **: P < 0.01, *: P < 0.05; n.s.: P > 0.05. Error bars denote SEM. BioRender was used to generate the illustration.

Despite reduced de novo purine synthesis, ETC blockade increases purine monophosphate abundance. In IACS-010759-treated cells, essentially 100% of the IMP pool is labeled as m+5 after 6 hours of culture with [U-^13^C]glucose (Figure S4B); this indicates that the entire IMP pool has turned over in 6 hours. However, under identical conditions, 6 hours of culture with [amide-^15^N]glutamine results in approximately 50% m+2 IMP fractional enrichment (Figure 4D), indicating that only 50% of the IMP that has turned over arises from de novo purine synthesis, with the rest presumably arising from purine salvage. To assess purine salvage, we first challenged cells with lometrexol (LTX) or methotrexate (MTX) to inhibit de novo purine synthesis, and traced with either [amide-^15^N]glutamine or [^15^N_4_]hypoxanthine (Figure 5A). As expected, both drugs suppressed de novo purine synthesis but stimulated purine salvage (Figure S5A and S5B). After 6 hours of culture with [^15^N_4_]hypoxanthine, IACS-010759-treated control cells displayed higher m+4 IMP enrichment than untreated cells, with NDI1 reversing this effect (Figure 5B). Kinetic tracing revealed increased labeling of both AMP and GMP from [^15^N_4_]hypoxanthine in IACS-010759-treated cells (Figure S5C). H460 cells deficient in LIPT1 also showed higher contribution of hypoxanthine to purine pools (Figure S4D and S4E). To ascertain whether purine salvage was necessary for purine nucleotide accumulation by IACS-010759, we generated H460 cells defective in the purine salvage enzyme hypoxanthine phosphoribosyl transferase 1 (HPRT1, Fig. S4F). Ablation of HPRT1 led to an elevation in intracellular hypoxanthine and nearly eliminated enrichment of m+4 purine nucleotides induced by IACS-010759 (Fig 5C and 5D). The accumulation of IMP, AMP, and GMP induced by IACS-010759 was also blunted in the absence of HPRT1 (Figure 5E), indicating that HPRT1-mediated purine salvage contributes to purine monophosphate accumulation upon ETC blockade. Neither HPRT1 expression nor its enzymatic activity in cell lysates was different between control and ETC-deficient cells (Figures S4G–H and S5G). Therefore we next tested whether enhanced purine salvage in ETC-deficient cells results from an increase in HPRT1 substrates.

### ETC blockade enhances the PPP

Purine salvage requires purine nucleobases and phosphoribosyl diphosphate (PRPP), an activated form of ribose-5-phosphate (R5P) produced in the pentose phosphate pathway (PPP). Therefore, we examined the effects of complex I inhibition on the PPP. R5P pools were depleted after 24 hours of IACS-010759 (Figure S5H). However, after addition of fresh medium containing [U-^13^C]glucose, both the abundance and m+5 labeling of R5P rose faster in IACS-010759-treated than DMSO-treated cells, indicating rapid synthesis of R5P from glucose during complex I blockade (Figures S5I to S5K). IACS-010759-treated cells also displayed higher enrichment of m+6 6-phosphogluconate (6-PG) and m+7 sedoheptulose 7-phosphate (S7P), two other PPP intermediates (Figure S5L), and more rapid labeling and higher abundance of PRPP (Figure 5F and 5G). Because the concentration of PRPP in human cells (~10 μM) is below its reported K_m_ for HPRT1 (approximately 200 μM)^35,36^, increasing PRPP abundance may facilitate purine salvage during ETC blockade.

To assess whether the oxidative or non-oxidative branch of the PPP predominated in these cells, we used [1,2-^13^C]glucose as a tracer. In this tracing scheme, both m+1 and m+2 R5P are produced, with m+1 arising predominantly from the oxidative branch and m+2 arising from the non-oxidative branch (Figure 5H). IACS-010759 treatment increased both m+1 and m+2 R5P abundance, but the fractional enrichment of m+1 increased while m+2 decreased in response to IACS-010759, and both were normalized by NDI1. (Figures 5I and 5J). NDI1 also eliminated the increase in PRPP m+5 induced by IACS-010759 (Figure 5K). Taken together, the data indicate an activation of PRPP synthesis, primarily through the oxidative branch of the PPP, in response to ETC blockade.

### HPRT1 is important for NSCLC growth especially when ETC is impaired

**Figure 6.**
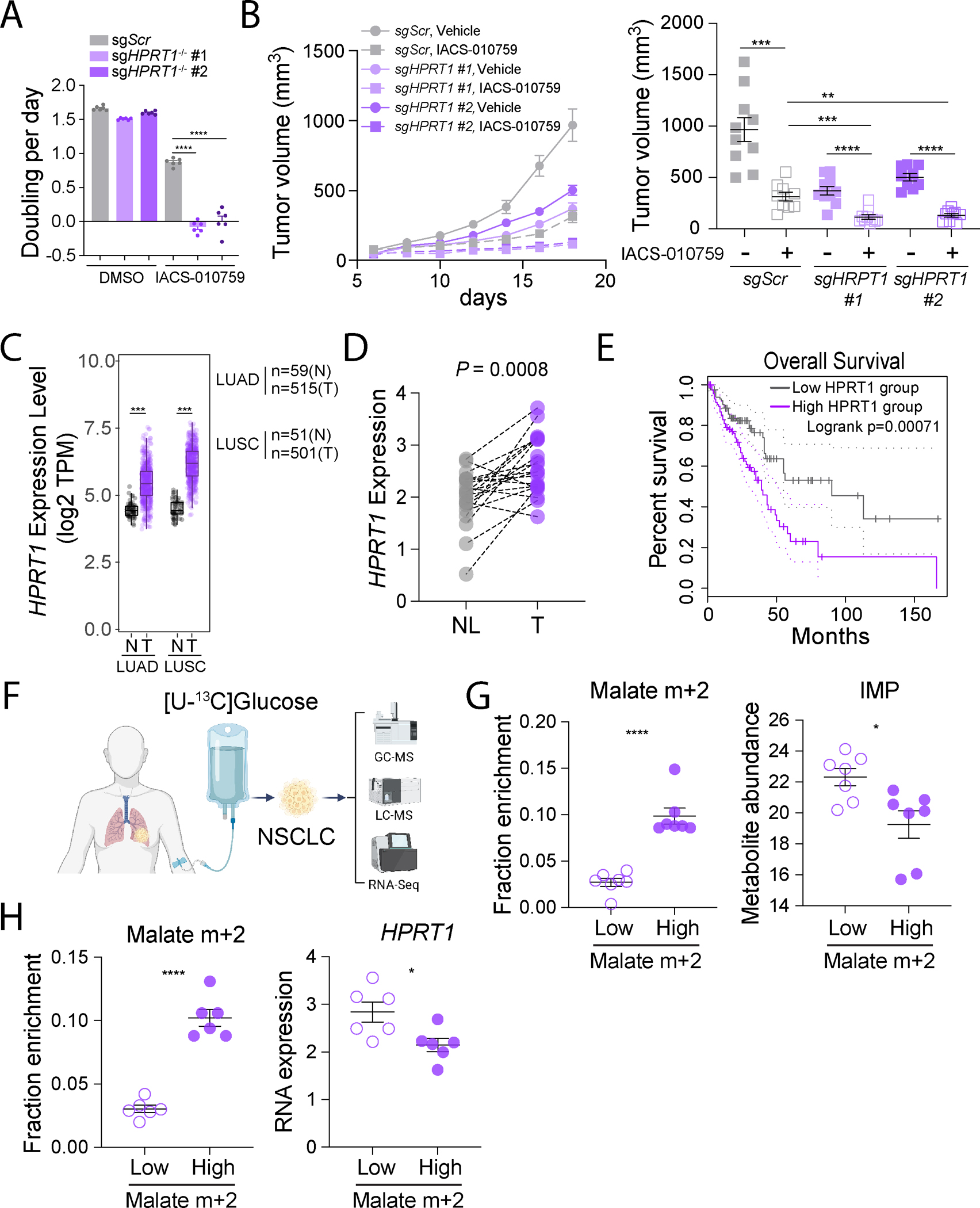
HPRT1 supports NSCLC growth during ETC inhibition. **A.** Cell growth rates of control and HPRT1-depleted H460 cells treated with DMSO or 25 nM IACS-010759 (n=6). Data are from one of three independent experiments. **B.** Subcutaneous growth of control and HPRT1-deficient H460 xenografts treated with vehicle or 5 mg/kg IACS-010759. The right panel shows individual tumor sizes on the day when the tumors were harvested. n=10 for *sgScr* Vehicle, *sgHPRT1* #1 Vehicle, and *sgHPRT1* #1 IACS-010759. n=9 for *sgScr* IACS-010759, *sgHPRT1* #2 Vehicle, and *sgHPRT1* #2 IACS-010759. **C.** *HPRT1* mRNA levels in human LUAD and LUSC tumors (T) or nonmalignant lung tissue (N). Data and statistics were generated using TIMER2.0^60,61^. **D.** Patient-matched *HPRT1* expression in human NSCLC tumors (T) and adjacent, nonmalignant lung (NL) (n=20). **E.** Kaplan-Meier plot showing overall survival of LUAD patients with high (top 25%, n=120) and low (bottom 25%, n=120) *HPRT1* expression. Hazard ratio (high) = 2.1. p(HR)=0.00097. The plot and statistics were generated using GEPIA 2^62^. **F.** Schematic illustrating intra-operative [U-^13^C]glucose infusion in patients with NSCLC followed by tumor resection and multi-omics analyses. **G.** Fractional enrichment of m+2 malate and relative IMP abundance in tumors displaying low or high malate labeling. The analysis was performed on the top and bottom 25% of tumors for malate m+2 labeling (n=7 tumors each with both isotope tracing and metabolomics analysis). **H.** Fractional enrichment of m+2 malate and *HPRT1* mRNA levels in tumors displaying low or high malate labeling. The analysis was performed on the top and bottom 25% of tumors for malate m+2 labeling (n=6 tumors each with both isotope tracing and RNA-Seq analysis). Unpaired, two-sided t tests (**A, B, G,** and **H**), and a paired t test (**D**) were used for the statistical analyses. ****: P < 0.0001; ***: P < 0.001; **: P < 0.01, n.s.: P > 0.05. Error bars denote SEM. BioRender was used to generate the illustration.

Since defective mitochondrial respiration inhibits de novo purine nucleotide synthesis, we hypothesized that purine salvage is essential for ETC-deficient cells. Indeed, compared to control cells, HPRT1-deficient cells are more sensitive to IACS-010759 (Figure 6A). Inhibiting de novo purine synthesis does not exacerbate the growth defect caused by ETC blockade (Figure S6A), indicating that ETC-deficient cells are less dependent on de novo purine synthesis for growth. To assess HPRT1’s role in purine metabolism in vivo, we subcutaneously injected control and HPRT1-deficient H460 cells into immunocompromised mice and dosed the mice with vehicle or IACS-010759. HPRT1 deficiency reduced tumor growth even without IACS-010759 treatment, but compared to the control tumors, HPRT1-deficient tumors were more sensitive to IACS-010759 (Figure 6B). These data indicate that although H460 cells tolerate HPRT1 loss in culture, this enzyme is required for maximal tumor growth in vivo and complex I blockade increases dependence on HPRT1-mediated purine salvage.

While de novo purine synthesis is the target of multiple chemotherapeutic drugs, the role of purine salvage in tumor growth remains underappreciated. Analysis of The Cancer Genome Atlas (TCGA) showed higher expression of *HPRT1* in human lung adenocarcinomas and squamous cell carcinomas compared to nonmalignant lungs (Figure 6C). We also observed enhanced *HPRT1* expression in NSCLCs relative to patient-matched lung tissue from our own cohort (Figure 6D). Moreover, high expression of *HPRT1* correlates with poor overall survival of patients with NSCLC (Figure 6E).

We next examined how mitochondrial function affects purine metabolism in human NSCLC in vivo. Intra-operative infusion of [U-^13^C]glucose during surgical NSCLC resection leads to variable labeling in TCA cycle intermediates extracted from the tumors (Figure 6F)^15,16^. In xenografts, ETC activity within NSCLC cells contributes to TCA cycle intermediate labeling from glucose^30^, so for this analysis we asked how labeling of these intermediates correlates with markers of purine metabolism. In human NSCLCs, there was a strong correlation between m+2 glutamate and m+2 malate, indicating label propagation around the TCA cycle (Figure S6B). Analysis of ^13^C labeling and metabolite abundance revealed that tumors with low malate m+2 enrichment contained more IMP (Figure 6G). RNA-sequencing revealed that tumors with low malate m+2 enrichment exhibited higher *HPRT1* expression (Figure 6H). Similar results were also obtained if we used m+2 glutamate for the analyses (Figure S6C and S6D). These data may indicate an enhanced propensity for purine salvage in human NSCLCs when glucose-dependent labeling of TCA cycle intermediates is low, as would be the case if OXPHOS is relatively impaired.

### Purine nucleotide accumulation induced by complex I inhibition is independent of macroautophagy

We next explored how cells acquire purine nucleobases for the salvage reaction. Cancer cells can use autophagy to generate purine nucleotides^37^. Some 80% of cellular RNA is ribosomal RNA, which accounts for most of the ribosomal mass^38,39^. The selective degradation of ribosomes by autophagy (ribophagy) contributes to nucleotide pools during nutrient starvation, and this process is negatively regulated by mTORC1^40,41^. Consistent with a previous study^29^, we observed repressed mTORC1 signaling upon IACS-010759 treatment, and this was restored by NDI1 (Figures S7A and S7B). Since mTORC1 inhibits ribophagy, we tested whether mTORC1 suppression induces ribosomal degradation and supplies nucleobases for purine salvage during IACS-010759 treatment. We generated an H460 ribophagy reporter cell line that expresses ribosomal protein 3 (RPS3) fused with a Keima-Red protein (Figure S7C). During ribophagy, RPS3-Keima-Red is cleaved to release Keima protein (Figure S7D, lower band)^40,42^. Treatment with the mTORC1 inhibitor Torin1 led to the expected cleavage of RPS3-Keima, and this was prevented by the autophagy inhibitor Bafilomycin A (Figure S7D). In contrast, IACS-010759 did not induce ribophagy (Figure S7D). IACS-010759 did decrease p62/SQSTM1, indicating augmented macroautophagy (Figure S7D). To examine whether macroautophagy contributes to purine accumulation, we generated H460 cells deficient in ATG5 or ATG7, two essential autophagy factors (Figure S7E). Deletion of ATG5 or ATG7 had no impact on the accumulation of IMP, GMP or AMP by IACS-010759 (Figure S7F), indicating that ETC blockade induces purine nucleotide accumulation independently of macroautophagy.

### ETC-deficient cells depend on nucleobase uptake to provide purine nucleotides for growth

Metabolic stress induces nutrient scavenging from the microenvironment to sustain cell survival and growth. ETC-deficient cells rely on environmental lipids for cell growth, and pancreatic cancer cells use macropinocytosis when aspartate synthesis is disrupted^43,44^. Of note, an unbiased CRISPR screen in pancreatic cancer cells identified both HPRT1 and SLC29A1, a nucleoside/base transporter, as conditionally essential during ETC blockade^44^. To investigate whether ETC-deficient cells rely on extracellular purine nucleobases for purine salvage and proliferation, we cultured cells in medium supplemented with either FBS or dialyzed FBS (dFBS). These two sera are metabolically different including much lower purine levels in dFBS (Figure S7G and S7H). Cells growing in dFBS-supplemented medium were more sensitive to ETC blockade, and growth was partially rescued by supplementing with purine nucleosides (Figure S7I). This led us to hypothesize that ETC-deficient cells take up nucleosides or nucleobases from the medium to supply the salvage pathway.

**Figure 7.**
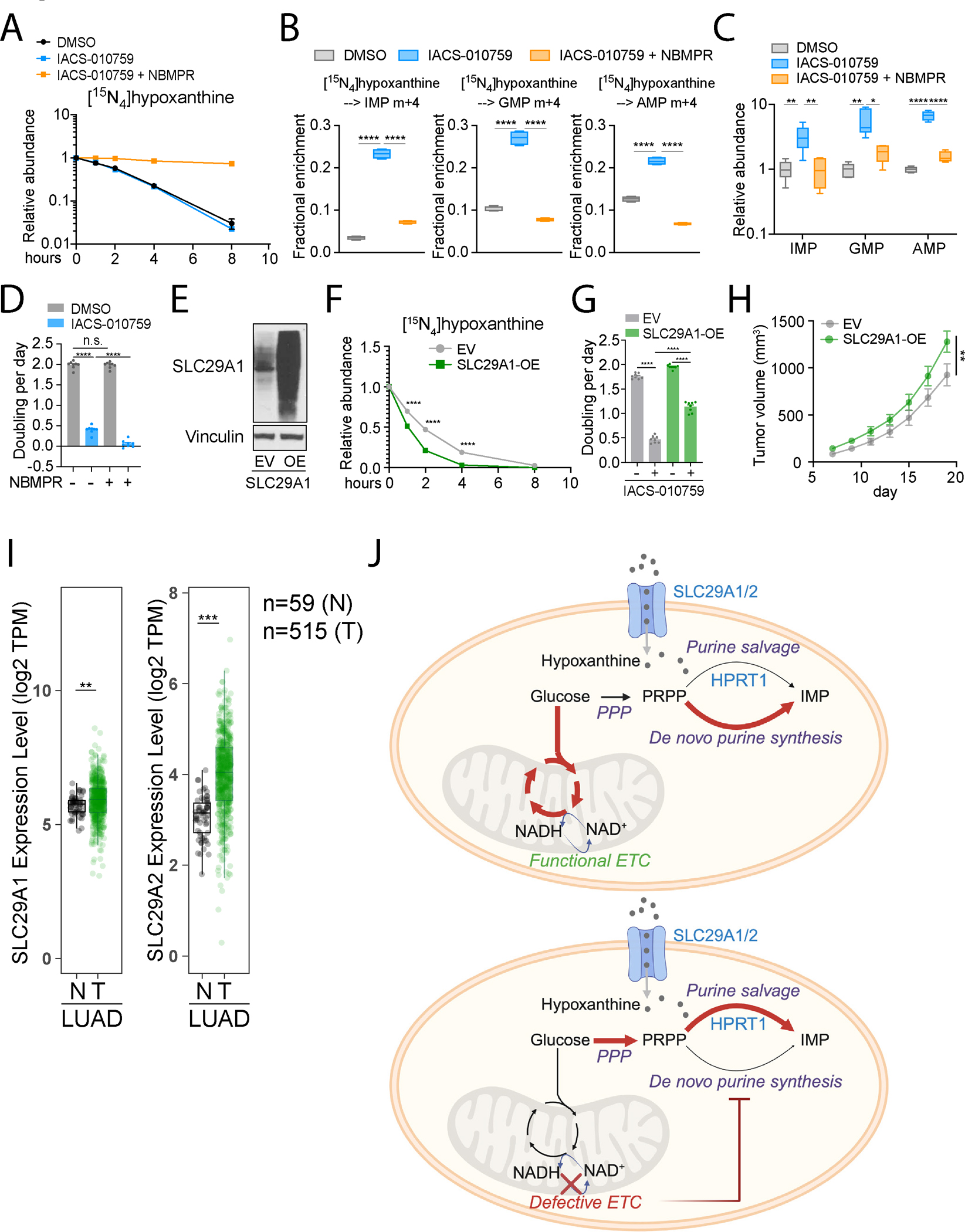
Purine uptake is required to supply salvage upon ETC blockade. **A.** Relative abundance of extracellular [^15^N_4_]hypoxanthine during 8 hours of culture of H460 cells treated with DMSO, 25 nM IACS-010759, or both 25 nM IACS-010759 and 50 μM NBMPR. **B.** Fractional enrichment of m+4 IMP, GMP, and AMP after 6 hours of culture with [^15^N_4_]hypoxanthine, following 24-hours of treatment with DMSO or 25 nM IACS-010759, with or without 50 μM NBMPR (n=3). **C.** Relative abundance of the indicated purine nucleotides in H460 cells after 24 hours of treatment with DMSO or 25 nM IACS-010759, with or without 50 μM NBMPR (n=6). **D.** Growth rates of H460 cells treated with 50 μM NBMPR, 25 nM IACS-010759, or both (n=8). Data are from one of three independent experiments. **E.** Western blot validating overexpression of SLC29A1. Vinculin is the loading control. EV: empty vector; OE: overexpression. **F.** Relative abundance of extracellular [^15^N_4_]hypoxanthine during 8 hours of culture of empty vector-expressing control cells (EV) and SLC29A1-overexpressing (SLC29A1-OE) H460 cells. **G.** Growth rates of control (EV) and SLC29A1-overexpressing (SLC29A1-OE) cells treated with DMSO or 25 nM IACS-010759 (n=8). Data are from one of three independent experiments. **H.** Tumor growth rates of control (EV) and SLC29A1-overexpression (SLC29A1-OE) H460 xenografts. n=14 for each group. **I.** *SLC29A1* and *SLC29A2* RNA levels in human lung adenocarcinoma (LUAD). N: nonmalignant lung; T: tumors. Data and statistics were generated using TIMER 2.0^60,61^. **J.** Reprogramming of purine synthesis pathways upon ETC suppression. Unpaired, two-sided t tests (**B-D**), and a two-way ANOVA test (**F** and **H**) were used for the statistical analyses. ****: P < 0.0001; ***: P < 0.001; **: P < 0.01, *: P < 0.05, n.s.: P > 0.05. Error bars denote SEM. BioRender was used to generate the illustration.

Two main nucleoside transporter groups, the SLC28 and SLC29 families, transport most purine nucleosides and nucleobases^45^. From RNA-seq data, we determined that H460 cells only express appreciable levels of SLC29A1 and SLC29A2 (Figure S7J). We treated H460 cells with DMSO, IACS-010759, or a combination of IACS-010759 and the SLC29A1/SLC29A2 inhibitor nitrobenzylthioinosine (NBMPR) and monitored consumption of unlabeled purine metabolites and [^15^N_4_]hypoxanthine from the medium. Hypoxanthine was rapidly depleted, but none of the other bases were taken up (Figures 7A and S7K). IACS-010759 did not potentiate hypoxanthine uptake, and NBMPR suppressed it (Figures 7A and S7K). Hypoxanthine uptake was dependent on HPRT1, because cells lacking HPRT1 displayed no net hypoxanthine consumption over time (Figure S7L and S7M). NBMPR reduced labeling of cellular purine nucleotides from [^15^N_4_]hypoxanthine (Figure 7B), and diminished purine nucleotide accumulation induced by IACS-010759 (Figure 7C). NBMPR did not alter proliferation of H460 cells under control conditions, but enhanced the effect of IACS-010759 (Figure 7D) and reduced proliferation in H460 cells lacking UQCRC2 (Figure S7N). These data indicate that ETC-deficient cells depend on extracellular hypoxanthine for purine salvage to sustain growth.

It is worth emphasizing that while total purine salvage contributes to approximately 50% of the IMP pool in IACS-010759-treated cells, the fractional enrichment of m+4 IMP was only around 20% after 6 hours of [^15^N_4_]hypoxanthine tracing (Figure 5B). These data indicate that approximately 30% of the IMP pool arises from HPRT1-dependent salvage reactions involving unlabeled bases. These unlabeled bases likley arise from purine recycling inside the cell, because unlabeled extracellular hypoxanthine is essentially absent in medium supplemented with dFBS (Figure S7H), including medium used in [^15^N_4_]hypoxanthine experiments. These results, along with the glucose and glutamine tracing data above indicate that the bases feeding purine salvage arise both inside and outside the cell, with rapid purine turnover contributing substantially to HPRT1-dependent purine salvage.

SLC29A1 overexpression promoted hypoxanthine uptake and blunted the effect of IACS-010759 on cell proliferation (Figures 7E–7G). SLC29A1 overexpression was also sufficient to enhance H460 xenograft growth, suggesting that purine nucleoside uptake is limiting in vivo for growth of these tumors (Figure 7H). In line with this, both *SLC29A1* and *SLC29A2* are more highly expressed in human lung adenocarcinoma relative to adjacent lungs, suggesting a role in human lung cancer (Figure 7I).

## DISCUSSION

We find that mitochondrial metabolism – specifically, the ability to engage in OXPHOS – dictates the pathway by which cells maintain pools of purines. ETC-deficient cells exhibit suppressed de novo purine synthesis and require purine uptake and salvage to maximize growth (Figure 7J). We observed alterations in purine metabolites in fibroblasts from patients with mitochondrial dysfunction, and in human NSCLCs where the contribution of glucose to the TCA cycle correlates inversely with markers of purine salvage. We also note that patients with cancer receiving IACS-010759 in a Phase I clinical trial exhibited elevated purine nucIeotides in the blood^46^, and that defects in mitochondrial DNA replication perturb purine-related metabolites in patients and mice^47^. These findings provide support for the disease relevance of our study.

Aspartate is required for the synthesis of SAICAR, a pivotal step in de novo IMP synthesis, and becomes limiting when the cell’s ability to recycle NADH to NAD^+^ is impaired^27,28^. Therefore, the suppression of de novo purine synthesis by ETC blockade is intuitively understandable. However, merely restoring intracellular aspartate is insufficient to restore de novo purine nucleotide synthesis in ETC-deficient cells, indicating the involvement of other factors in suppressing de novo purine synthesis under these conditions. For example, ETC dysfunction also disrupts one-carbon metabolism which provides N^10^-formyl-tetrahydrofolate for de novo purine synthesis pathway^48^.

An intriguing aspect of the data is that ETC inhibition not only results in a switch from de novo purine synthesis to purine salvage to maintain purine monophosphate pools, but also expands these pools. We speculate that accumulation of purine monophosphates is an adaptive response to help cells cope with compromised ETC function. De novo purine nucleotide synthesis from PRPP to IMP is energetically demanding, requiring contributions from glutamine, glycine, aspartate, and N^10^-formyl-tetrahydrofolate, and is subject to feedback inhibition by purine monophosphates^49,50^. Constitutive de novo purine synthesis would be counterproductive and perhaps toxic when the cell’s ability to produce and maintain a favorable energy state and pools of required intermediates is insufficient to complete the pathway^51^. The accumulated purine monophosphates may facilitate inhibition of PPAT^52^, which catalyzes the committed step of the de novo pathway, therefore preventing unnecessary energy expenditure.

The low NAD^+^:NADH ratio induced by ETC dysfunction is a key factor in elevated IMP and hypoxanthine levels. While previous studies have touched on the association between redox imbalance and IMP accumulation in cells with defective OXPHOS^28,53–55^, our study further delineates the distinct roles of compartmentalized NAD^+^:NADH ratios in modulating metabolic responses to ETC blockade. Additional evidence linking purine accumulation to excess NADH includes the observation that expressing the *Escherichia coli* pyridine nucleotide EcSTH in HeLa cells reduces the NAD^+^:NADH ratio while increasing the abundance of several purines, and that ethanol ingestion decreases the NAD^+^:NADH ratio and induces purine monophosphate accumulation in the mouse liver^56^. Collectively, these observations indicate an important role for the NAD^+^:NADH ratio in regulating the mode of purine metabolism.

The balance between de novo purine synthesis and purine salvage is particularly relevant in cancer, where mitochondrial function is variable and drugs can be used to inhibit either pathway. In some tumors, mutations in mitochondrial enzymes may render cells permanently reliant on purine salvage. Recent data demonstrate that fumarate hydratase (FH)-deficient renal carcinoma cells have suppressed de novo purine synthesis and require purine salvage^57^. We note that H460 cells, which respire well and do not require HPRT1 for growth in culture, nevertheless require this enzyme for maximal growth of subcutaneous xenografts. These findings indicate that dependence on purine salvage can be imposed on respiration-competent cells by environmental factors. Rapidly growing tumors also experience hypoxia, which may further enhance salvage dependence^34,58,59^. This may also explain why NSCLCs tend to over-express *HPRT1*, *SLC29A1,* and *SLC29A2*. The fact that over-expressing *SLC29A1* is sufficient to drive xenograft growth argues that access to purine nucleosides or nucleobases is a limiting factor for H460 cell growth in vivo.

### Limitations of the study

Low metabolite abudnace and the limitations of LC/MS resolution prevented us from detecting all purine intermediates and pinpointing the exact mechanism by which ETC blockade suppresses de novo purine synthesis. We also have not fully defined the signals that regulate the switch from the de novo purine synthesis to purine salvage when mitochondrial respiration is compromised. Although the NAD^+^:NADH ratio is involved, neither Cyto- nor Mito-*Lb*NOX expression in *UQCRC2*^*−/−*^ cells increased this ratio to the level observed in parental cells, and we lack tools to precisely measure the NAD^+^:NADH ratio in a compartment-specific manner. This leaves the possibility that other aspects of ETC function beyond redox maintenance regulate purine salvage. Finally, it is interesting that ETC inhibition does not enhance hypoxanthine uptake in the assays we used, given that ETC inhibition stimulates HPRT1-dependent salvage and HPRT1 stimulates hypoxanthine import. Given that SLC29A transporters are equilibrative, it may be that ETC blockade also increases the pool of intracellular hypoxanthine such that no increase in net import occurs. Further quantitative analysis of hypoxanthine uptake and metabolism may help resolve this issue.

## STAR METHODS

### Lead Contact

Further information and requests for resources and reagents should be directed to the lead contact, Ralph DeBerardinis, MD, PhD.

Ralph.DeBerardinis@utsouthwestern.edu

### Material Availability

Isogenenic cell lines and DNA constructs generated in this paper are available upon request.

### Data and Code Availability:

The raw RNA-seq data reported in this study have been deposited in Gene Expression Omnibus (GEO) (GSE265923). The raw proteomics data in this study have been deposited in MassIVE (MSV000094553). Accession numbers are also listed in the key resource table. An Excel file containing the values to create graphs in the paper and a PDF file containing uncropped scans of all western blots are provided as Data S1. Raw metabolomics data are provided as Data S2. R script used to analyze the data can be found on the GitHub repository (https://github.com/wencgu/nac).

### Cell culture

H460 and H157 cells were obtained from the Hamon Cancer Center Collection (University of Texas Southwestern Medical Center) and maintained in RPMI-1640 (Thermo Fisher Scientific, CB-40234) supplemented 10% fetal bovine serum (FBS). 293T, SK-N-DZ, A549 and 786-O cells were from American Type Culture Collection (ATCC, CRL-3216; CRL-2149; CCL-185; CRL-1932). 293T and 786-O cells were maintained in high glucose DMEM with 10% FBS. A549 and SK-N-DZ cells were maintained in RPMI-1640 with 10% FBS. Patient-derived fibroblasts were cultured in low glucose DMEM (Sigma, D6046) supplemented with 5% heat inactivated FBS. All cells were cultured at 37°C in a humidified atmosphere with 5% CO_2_.

### Clinical samples

All patients provided informed consent. Both the inborn errors of metabolism study (NCT02650622) and the lung cancer study (NCT02095808) were approved by the Institutional Review Board (IRB) at University of Texas Southwestern Medical Center (UTSW). For the inborn errors of metabolism study, plasma samples were obtained from fresh blood collected in heparinized tubes at the Children’s Medical Center at Dallas. Punch biopsies of the skin for fibroblast culture were obtained from the patient, following standard culture procedures for clinical diagnostics. For the lung cancer study, patients were infused with [U-^13^C]glucose and samples were obtained as described^15^.

### Gene deletion and over-expression

To generate *UQCRC2*^*−/−*^ H460 cells, cells were transfected with the PX458 construct^63^, a gift from Feng Zhang (Addgene plasmid #48138; http://n2t.net/addgene:48138; RRID:Addgene_48138) that contains sgRNA against human *UQCRC2*. Cells with the highest 2–5% GFP signal were selected two to three days after transfection using a flow cytometer (BD FACSAria II). Single cells were cultured in RPMI with 10% FBS, 1mM sodium pyruvate, 100 μg/mL uridine and penicillin/streptomycin. Loss of UQCRC2 protein was validated by western blot. To generate sgScr, sgHPRT1, sgATG5, and sgATG7 H460 cells, the indicated gRNAs were cloned into the LentiCRISPRv2 vector^64^, a gift from Feng Zhang (Addgene plasmid # 52961; http://n2t.net/addgene:52961; RRID:Addgene_52961) and transfected into 293T cells using Lipofectamine 3000 (Thermo Fisher Scientific L3000015) with a 2:1 ratio of psPAX2:pMD2G. The same method was used to generate RPS3-Keima cells using pLENTI_RPS3_Keima construct, a gift from Thomas Tuschl (Addgene plasmid # 127140; http://n2t.net/addgene:127140; RRID:Addgene_127140). For NDI1, *Lb*NOX, SLC1A3, and SLC29A1 overexpression, we utilized the PMXS-IRES-Bsd retroviral expression vector. The PMXS-NDI1^65^ was a gift from David Sabatini (Addgene plasmid # 72876; http://n2t.net/addgene:72876; RRID:Addgene_72876). The PMXS-SLC1A3^27^ was a gift from David Sabatini (Addgene plasmid # 72873; http://n2t.net/addgene:72873; RRID:Addgene_72873). PMXS-Cyto-*Lb*NOX and PMXS-Mito-*Lb*NOX vectors were a gift from Kivanc Birsoy. To generate stable gene-overexpressing H460 cell lines, the indicated PMXS constructs were transfected into 293T cells using Lipofectamine with a 2:1 ratio of Gag-Pol:VSVG. After 48 hours of transfection, medium containing viral particles was harvested and filtered using 0.45μm membranes and immediately used to culture H460 cells in the presence of 4 μg/mL polybrene (Sigma, TR-1003-G). After 24 hours, cells were subjected to 2 μg/mL puromycin (Thermo Fisher Scientific, NC9138068) or 10 μg/mL blasticidin (Thermo Fisher Scientific, NC1366670) selection until all the uninfected cells died. DNA oligos were purchased from IDT and contained the following sequences:

**Table:** 

sgRNA	Sequence
UQCRC2	5’- GCAAAGGCCAACTACCGTGG -3’
HPRT1 #1	5’- TTATGGCGACCCGCAGCCC -3’
HPRT1 #2	5’- TCTTGCTCGAGATGTGATGA -3’
ATG5 #1	5’- TGATATAGCGTGAAACAAGT -3’
ATG5 #2	5’- ATCACAAGCAACTCTGGAT -3’^42^
ATG7 #1	5’- CCCGTTGCTGCCCAGCTAT -3’
ATG7 #2	5’- TCCAAGGCACTACTAAAAG -3’^42^
Scrambled (Scr)	5’- TTCTTAGAAGTTGCTCCACG -3’

### NAD^+^ and NADH quantitation

Qualitative analysis of NAD^+^ and NADH was performed as previously described^66^ on a QExactive HF-X mass spectrometer (Thermo Scientific, Bremen, Germany). We perfomed quantitative analysis of NAD^+^ and NADH according to our previous protocol^67^ on a 6500+ mass spectrometer (AB Sciex, Framingham, MA). To prepare quantitative samples, cells were washed with saline and extracted with 40:20:20 acetonitrile:methanol:water (v/v) and 0.1 M formic acid and then neutralized with 15% ammonium bicarbonate (w/v). A ^15^N_5_-AMP internal standard was added to each extract at the final concentration of 100 nM. Samples were run the same day to minimize oxidation of the analytes of interest.

All cellular extracts were analyzed against an 8-point standard curve ranging from 5 nM to 1000 nM. All standard curves had R^2^ values greater than or equal to 0.98 with greater than 6 calibrators having accuracies within 20% of their known concentration.

### Targeted metabolomics

To extract metabolites, cells were rinsed with ice-cold saline twice and quenched by 80% cold methanol. Cells were incubated at −80°C for at least 20 minutes and then scraped. For tumor samples, the tissues were thawed and homogenized in cold 80% methanol using plastic pestles (Thermo Fisher Scientific, 12141364). Samples were subjected to three freeze-thaw cycles in liquid nitrogen and a 37°C water bath. Afterwards, the samples were vortexed for 1 minute and spun down at 4°C at 20,160 x g for 15 minutes. The supernatants were transferred into fresh Eppendorf tubes and dried in a SpeedVac concentrator overnight. To measure metabolites from conditioned media, 10 μL of medium was collected and added into 100 μL of 80% methanol followed by vortexing. The samples were then dried in a SpeedVac concentrator overnight.

Metabolite abundance was analyzed using multiple mass spectrometers. For analysis on a Q-TOF mass spectrometer, dried metabolites were reconstituted in 0.1% formic acid in analytical-grade water and vortexed for 1 minute before spinning at 4°C at 20,160 x g for 15 minutes. The supernatants were transferred to auto-sampler vials. Data acquisition was performed by reverse-phase chromatography on a 1290 UHPLC liquid chromatography (LC) system interfaced to a 6550 iFunnel Q-TOF mass spectrometer (MS) (Agilent Technologies, CA). The MS was operated in both positive and negative (ESI+ and ESI−) modes. Analytes were separated on an Acquity UPLC^®^ HSS T3 column (1.8 μm, 2.1 × 150 mm, Waters, MA). The column was kept at room temperature. Mobile phase A composition was 0.1% formic acid in water and mobile phase B composition was 0.1% formic acid in 100% ACN. The LC gradient was 0 min: 1% B; 5 min: 5% B; 15 min: 99%; 23 min: 99%; 24 min: 1%; 25 min: 1%. The flow rate was 250 μL min^−1^. The sample injection volume was 5 μL. ESI source conditions were set as follows: dry gas temperature 225°C and flow 18 L min^−1^, fragmentor voltage 175 V, sheath gas temperature 350°C and flow 12 L min^−1^, nozzle voltage 500 V, and capillary voltage +3500 V in positive mode and −3500 V in negative. The instrument was set to acquire over the full *m/z* range of 40–1700 in both modes, with the MS acquisition rate of 1 spectrum s^−1^ in profile format.

Raw data files (.d) were processed using Profinder B.08.00 SP3 software (Agilent Technologies, CA) with an in-house database containing retention time and accurate mass information on 600 standards from Mass Spectrometry Metabolite Library (IROA Technologies, MA). The in-house database matching parameters were: mass tolerance 10 ppm; retention time tolerance 0.5 min. Peak integration results were manually curated in Profinder for improved consistency and exported as a spreadsheet (.csv).

Samples prepared for analysis on a Q-Exactive were reconstituted in 80% acetonitrile and centrifuged at 4°C at 20,160 x g to remove insoluable material. Chromatographic separation of metabolites was carried out on a Vanquish UHPLC system equipped with a ZIC-pHILIC column (Millipore-Sigma, Burlington, MA) as previously described^66,68,69^. Extracted ion chromatograms (XICs) were generated with a mass tolerance of 5 ppm and integrated for relative quantitation. Identities of analytes were confirmed with purified standards and product ion spectra.

Principal component analyses and metabolite set enrichment analyses were conducted using MetaboAnalyst 5.0^70^.

### Stable isotope tracing

For tracing with ^13^C-glucose, cells were cultured in base RPMI medium (Sigma, R1383-L) supplemented with 11 mM [U-^13^C]glucose (Cambridge Isotope Laboratories, CLM481–0.25) or [1,2-^13^C]glucose (Cambridge Isotope Laboratories, CLM-504–0.5) and 10% dialyzed FBS (Gemini Bio-Products, 100108). For tracing with [amide-^15^N]glutamine, cells were cultured in glutamine-free RPMI medium (Sigma, R0883) supplemented with 2 mM [amide-^15^N]glutamine (Cambridge Isotope Laboratories, NLM-557–1) or 2 mM [U-^13^C]glutamine (Cambridge Isotope Laboratories, CLM-1822–0) and 10% dialyzed FBS. For tracing with [^15^N_4_]hypoxanthine, cells were cultured in RPMI medium containing 10% dialyzed FBS and 10 μM [^15^N_4_]hypoxanthine (Cambridge Isotope Laboratories, NLM-8500–0.1). For all drug-treated samples, cells were exposed to the drug during tracing. The metabolites were extracted as described in the targeted metabolomics method. For analysis by Q-TOF, data acquisition was performed and analyzed according to the methods described above.

For analysis on the Q-Exactive, tSIM methods were used to increase the signal of isotopically-labeled intermediates of the purine biosynthetic pathway and pentose phosphate pathway. Both ^15^N and ^13^C nuclei were analysed using this approach. Quadrupole isolation windows for individual analytes were set to capture all relevant nuclei to calculate fractional enrichment values. We performed analysis of isotopologues according to our previously reported method^71^. Natural isotope abundances were corrected using a customized R script, which can be found at the GitHub repository (https://github.com/wencgu/nac). The script was written by adapting the AccuCor algorithm^72^.

For targeted analysis of purines and hypoxanthine tracing using AB SCIEX QTRAP 5500 LC/triple quadrupole MS (Apllied Biosystems SCIEX), metabolites were reconstituted in 0.1% formic acid in analytical water, vortexed, and spun down to remove insoluble material before being loaded onto the instrument as previously described^73^. Using a Nexera Ultra-High-Performance Liquid Chromatograph system (Shimadzu Corporation), we achieved separation on a Waters Symmetry C18 column (150 × 2.1 mm, 3.5um) with 0.1 % formic acid in mobile phase A (H_2_O) and mobile phase B (acetonitrile) in a flow rate at 0.4 mL with injection volume at 10 μL. The gradient elution is 0–5 min, 0–30% B; 5–6 min, 30–100% B; 6–8 min, 100% B; 8–9 min, 100–0% B; 9–10 min, 12% B. Chromatogram review and peak area integration were performed using MultiQuant (version 2.1, Applied Biosystems SCIEX). The MRMs used are listed as follows: AMP Q1/Q3 (348/136 (m+0), 352/140 (m+4), CE: 30); IMP Q1/Q3 (349/137 (m+0), 353/141 (m+4), CE: 22); GMP Q1/Q3 (364/152 (m+0), 368/156 (m+4), CE: 18); Hypoxanthine Q1/Q3 (137/119 (m+0), 141/123 (m+4), CE: 27) or (137/110 (m+0), 141/113 (m+4), CE: 27).

### Gas chromatography/mass spectrometry (GC/MS)

Metabolites were extracted as described in the targeted metabolomics method and 1 μL D_27_-myristic acid was added as an internal control. The dried metabolites were re-suspended in 40 μL anhydrous pyridine and transferred to GC/MS autoinjector vials. The samples were incubated at 70°C for 15 min, followed by addition of 80 μL N-(tert-butyldimethylsilyl)-N-methyltrifluoroacetamide (MTBSTFA) derivatization reagent, as previously described^15^. The samples were incubated at 70°C for 1 hour before being subjected to GC-MS analysis. 1 μL of the sample was injected for analysis on Agilent 6890 or 7890 gas chromatographs coupled to an Agilent 5973N or 5975C Mass Spectrometer. The data were analyzed using EL-MAVEN, and observed distributions of mass isotopologues were corrected for natural abundance using a customized R script on the GitHub repository (https://github.com/wencgu/nac).

### HPRT1 enzymatic activity analysis

HPRT1 enzyme activity was measured using the Precise HPRT1 assay kit (Novo CIB, K0709–01-2) according to manufacturer’s instructions. In brief, H460 cells were seeded in 10 cm plates and treated with DMSO or 25 nM IACS-010759 for 24 hours. Cells were rinsed once with PBS, scraped, and lysed in ice-cold lysis buffer containing 10 mM Tris-HCl pH 7.4, 150 mM NaCl, 1% NP-40, 1 mM EDTA followed by centrifugation at 18,000 x g for 10 minutes at 4°C. Each enzymatic reaction contained 5 μL sample or positive control (human recombinant HPRT enzyme) and 200 μL reaction mixture containing DTT (cofactor 1), NAD (cofactor 2) and bacterial IMPDH in the absence (blank) or presence of 2 mM PRPP (enzyme reaction). The reaction was performed at 37°C. The absorbance at 340 nm was recorded at 2-minutes intervals for 120 minutes. HPRT1 protein abundance in each sample was assessed by immunoblotting using antibody against HPRT1. Image J was used to quantify the HPRT1 band intensity. HPRT1 catalytic rate was normalized to the protein abundance in each group with the DMSO control samples given a value of 1.

### Glucose uptake and lactate secretion assay

Cells were seeded in 6 cm plates. The glucose uptake and lactate secretion analysis was started when cells reached 90% confluence. Cells were washed with PBS once. 2 mL medium containing 25 nM IACS-010759 or equal volume of DMSO was added into the plates for 6 hours. Medium from each plate was collected and spun down at 20,160 x g at 4°C. 1 mL supernatant of each sample was transferred to a fresh eppendorf tube and loaded in a NOVA instrument to measure glucose and lactate levels. Three or four tubes containing medium but no cells were used as blanks to calculate the amount of glucose and lactate taken up or secreted by cells. The cell number was counted from each plate to calculate the rate of glucose uptake and lactate secretion per cell.

### Cell growth analysis

Cells were seeded in flat, clear bottom 96 well plates. Cells were stained with 5 μg/mL Hoechst (Thermo Fisher Scientific, 62249) and 1 μg/mL Propidium Iodide (PI) (Thermo Fisher Scientific, P3566) in PBS for at least 15 minutes at 37°C and then subjected to cell counting using a Celigo Imaging Cytometer. Live cells were calculated as the total number of Hoechst-positive cells minus the number of PI-positive cells. Proliferation rate was calculated as previously described^23^. Chemicals added to the medium for the growth assay were: IACS-010759 (25nM, ChemieTek, CT-IACS107), pyruvate (1mM, Sigma-Aldrich, S8636), uridine, AKB (1mM, Sigma-Aldrich, K0875–5G), inosine (50μM, Millipore Sigma, I4125), guanosine (50μM, Millipore Sigma, G6752), adenosine (50μM, Millipore Sigma, A9251), and NBMPR (50μM, N2255, Millipore Sigma), nocodazole (100 nM, Fisher Scientific, 50–194-7956).

### Immunoblotting

Cells were washed with PBS and then lysed in RIPA buffer (Boston BioProducts, BP-115) containing proteinase and phosphatase inhibitors (Thermo Fisher Scientific, 78444). To blot the transporter proteins, cells were lysed in buffer containing 10 mM Tris-HCl (pH 7.5), 150 mM NaCl, 1 mM EDTA, 1% Trion X-100, 2% SDS, and 0.1% CHAPS, followed by sonication. Samples were spun down at 4°C at 20,160 x g for 10 minutes and supernatants were collected for protein measurement using the DC Protein Assay Kit (Bio-Rad, 5000111). Equal amounts of protein were loaded to run the gels (Thermo Fisher Scientific, NP0323BOX) and then transferred to PVDF membrane (Thermo Fisher Scientific, 88518). Membranes were dipped in methanol for 20 seconds and then rinsed with DI water. The air-dried membrane was incubated with primary antibodies in filtered PBS containing 5% BSA and 0.1% Tween-20 (PBST) at 4°C overnight. The membranes were washed with PBS 3 times for 5 minutes and then incubated with horseradish peroxidase conjugated secondary antibody (Cell Signaling Technology, 7074, 7076) in 5% non-fat milk in PBST at room temperature for 1 hour. Membranes were washed 5 times for 5 minutes with PBS at room temperature and then exposed to Pierce ECL (Thermo Fisher Scientific, PI32106) for 2 minutes. Signals were detected using either Amersham imagequant 800 or films in the dark room. Antibodies used for western blots are: Vinculin (Proteintech, 26520–1-AP), UQCRC2 (Abcam, ab14745), SLC1A3 (Cell Signaling Technology, #4166), HPRT1 (Santa Cruz Biotechnology, sc-376938), SLC29A1 (Abcam, ab182023), p70 S6 Kinase (S6K) (Cell Signaling Technology, 9202S), Phospho-p70 S6 Kinase P-S6K (Thr389) (Cell Signaling Technology, 9234S), 4E-BP1 (Cell Signaling Technology, 9644S), Phospho-4E-BP1 (Ser65) (Cell Signaling Technology, 9451S), GAPDH (Cell Signaling Technology, 2118S), RPL26 (Bethyl Laboratories, A300–685A-T), Keima (MBL International, M1823B), LC3B (Sigma-Aldrich, L8918), p62 (Cell Signaling Technology, 88588S), ATG5 (Cell Signaling Technology, 9980S), ATG7 (Cell Signaling Technology, 8558T).

### Xenograft studies in mice

All mouse experiments complied with relevant ethical regulations and were performed according to protocols approved by the Institutional Animal Care and Use Committee at the University of Texas Southwestern Medical Center (protocols 2016–101360 and 2016–101694). H460 cells were suspended in serum-free RPMI medium and mixed with Matrigel (Thermo Fisher Scientific, CB-40234) at 1:1 volume ratio. One million cells were subcutaneously injected into the right flank of NOD.Cg-*Psrkdcscid Il2rgtm1Wjl*/SzJ (NSG) mice. Mice were randomized for IACS-010759 treatments and then administered vehicle or IACS-010759 daily through oral gavage (5 or 10mg/kg body mass in 100 μL of 0.5% methylcellulose and 4% DMSO)^26,30^. For tumor metabolomics and glutamine infusion experiments, mice were dosed with (10mg/kg) IACS-010759 for 5 days prior to the infusion as previously described^30^. After the infusion, tumors were collected and snap frozen for later metabolite extraction and LC-MS analyses. For tumor growth analyses, mice were administered 5 mg/kg IACS-010759 until the day before sacrifice. Two orthogonal measurements of tumor diameter were collected every other day and tumor volume was calculated using the formula V = (L_1_x(L_2_^2^))/2.

### [amide-^15^N]glutamine infusion

Mice were anesthetized using (30 mg/mL) ketamine/xylazine mix (30 μL/g). 25-gauge catheters were placed in the lateral tail vein under anesthesia. The total dose of glutamine was 1.725 g/kg dissolved in 1.5 mL saline. Isotope infusions started with 150 μL/minute bolus for 1 minute followed by continuous infusion at rate of 150 μL/hour for 4 hours. Upon termination of the infusions, animals were euthanized immediately and tumors were collected and snap frozen in liquid nitrogen.

### Seahorse XFe96 Respirometry

An XFe96 Extracellular Flux Analyzer (Agilent Technologies) was used to measure oxygen consumption rate. In brief, 20,000 cells per well were seeded and simultaneously treated with 25 nM IACS-010759. After 16 to 20 hours, cells were washed three times with Seahorse medium (Agilent Technologies, 102353) containing 2 mM glutamine, 1 mM pyruvate, 10 mM glucose and pen/strep (pH 7.4) and incubated in a CO_2_-free incubator at 37°C for at least 30 minutes prior to loading into the instrument. Final concentrations for oligomycin A, carbonyl cyanide m-chlorophenylhydrazone (CCCP), and rotenone were 2 μM, 1 μM, and 2 μM respectively. After the assay, cells were counted using Celigo Image Cytometer (see method: cell growth analysis) to normalize oxygen consumption rate.

### Immunofluorescence and confocal microscopy

Coverslips were coated with 10 μg/mL fibronectin (Sigma-Aldrich, F1141–5MG) for 1 hour at 37°C and rinsed once with PBS. Cells were immediately seeded on the coverslips. Cells were fixed the next day with fresh warm 4% paraformaldehyde (PFA) solution in PBS for 15 minutes followed by permeabilization using 0.1% (v/v) Triton X-100 in PBS at room temperature for 10 minutes. Cells were then blocked in filtered PBS containing 1% BSA for at least 30 minutes at room temperature before incubation with primary antibodies against FLAG (1:200, F1804, Sigma-Aldrich) and HSP60 (1:500, 12165S, CST) for 1 hour at room temperature. Cells were washed 3 times for 5 minutes with PBS and then incubated with secondary antibodies (Alexa fluorophores 488 and 555, Invitrogen) for 1 hour in dark at room temperature. Coverslips were washed with PBS 3 times for 5 minutes and Mili-Q water once before being mounted on slides by Profade-Antifade (P36935, Invitrogen) overnight in dark. Cells were imaged using Zeiss LSM 880 Confocal Laser Scanning Microscope with Z-stacks acquired. The images labeled “Merge” are composites. All representative images were processed using Image J.

### RNA-seq

RNA was extracted using Trizol (Thermo Fisher Scientific, 15596018) and an RNeasy Mini Kit (Qiagen, 74106). A Qubit fluorometer and Invitrogen Qubit RNA High Sensitivity kit (Invitrogen, Q32852) were used to measure total RNA levels. RNA-seq libraries were prepared using the NEBNext Ultra II directional RNA library prep kit with the NEBNext Poly(A) mRNA magnetic isolation module (New England Biolabs, E7490L, E7760L) according to manufacturer’s instructions. Libraries were stranded using standard N.E.B indices according to manufacturer’s instructions (New England Biolabs, E7730L, E7335L, E7500L). Sequencing reads from all RNA-seq experiments were aligned to hg19 reference genome by STAR v. 2.5.2b^74^ with the following parameters: --runThreadN 28 --outSAMtype BAM SortedByCoordinate --outFilterMultimapNmax 1 --outWigStrand Unstranded --quantMode TranscriptomeSAM. Output BAM files were converted to BED format using the “bamtobed” command from BEDtools v.2.29.2 [https://bedtools.readthedocs.io/en/latest/]. BED files were then converted to a normalized wiggle file using a custom python script. Normalized wiggle files were then converted to bigwig format using wigToBigWig with “-clip” parameter. Read counts were derived using HTSeq^75^ with parameter “-s no” and 1 additional read count was added to each gene for each independent sample prior to downstream analyses. Differentially expressed genes were identified by DESeq2 (fold change ≥ 1.5, FDR-adjusted P value ≤ 0.05)^76^. Fragments Per Kilobase Of Exon Per Million Fragments Mapped (FPKM) value of genes were calculated by normalizing the gene length and sequencing depth.

### Quantitative proteomics

H460 cells were treated with 25 nM IACS-010759 or DMSO for 24 hours. To isolate protein, cells were washed twice with ice-cold PBS followed by addition of freshly prepared lysis solution consisting of 5% SDS in 50 mM TEAB with protease and phosphatase inhibitors. Cells were scraped in lysis solution and transferred to 1.5 mL Eppendorf tubes. Samples were allowed to sit at room temperature for 15 minutes to complete lysis. Protein concentration was calculated with a BCA assay and all samples were normalized to the same protein concentration. Following disulfide bond reduction and alkylation, samples were digested overnight with trypsin using an S-Trap (Protifi). The peptide eluate from the S-Trap was dried and reconstituted in 100 mM TEAB buffer. A TMT10plex Isobaric Mass Tagging Kit (Thermo) was used to label the samples as per the manufacturer’s instructions. The combined sample then underwent solid-phase extraction cleanup with an Oasis HLB plate (Waters) and was dried in a SpeedVac. The sample was then reconstituted in a 2% acetonitrile, 0.1% TFA buffer and diluted such that ~1 ug of peptides were injected.

Peptides were analyzed on a Thermo Orbitrap Eclipse MS system coupled to an Ultimate 3000 RSLC-Nano liquid chromatography system. Samples were injected onto a 75 um i.d., 75-cm long EasySpray column (Thermo) and eluted with a gradient from 0–28% buffer B over 180 minutes at a flow rate of 250 nL/minute. Buffer A contained 2% (v/v) ACN and 0.1% formic acid in water, and buffer B contained 80% (v/v) ACN, 10% (v/v) trifluoroethanol, and 0.1% formic acid in water. at a flow rate of 250 nL/minute. Spectra were continuously acquired in a data-dependent manner throughout the gradient, acquiring a full scan in the Orbitrap (at 120,000 resolution with a standard AGC target) followed by MS/MS scans on the most abundant ions in 2.5 s in the ion trap (turbo scan type with an intensity threshold of 5,000, CID collision energy of 35%, standard AGC target, maximum injection time of 35 ms and isolation width of 0.7 m/z). Charge states from 2–6 were included. Dynamic exclusion was enabled with a repeat count of 1, an exclusion duration of 25 s and an exclusion mass width of ± 10 ppm. Real-time search was used for selection of peaks for SPS-MS3 analysis, with searched performed against the human reviewed protein database from UniProt. Up to 1 missed tryptic cleavage was allowed, with carbamidomethylation (+57.0215) of cysteine and TMT reagent (+229.1629) of lysine and peptide N-termini used as static modifications and oxidation (+15.9949) of methionine used as a variable modification. MS3 data were collected for up to 10 MS2 peaks which matched to fragments from the real-time peptide search identification, in the orbitrap at a resolution of 50,000, HCD collision energy of 65% and a scan range of 100–500.

Protein identification and quantification used Proteome Discoverer v.3.0 SP1 (Thermo). Raw MS data files were analyzed against the human reviewed protein database from UniProt. Both Comet and SequestHT with INFERYS Rescoring were used, with carbamidomethylation (+57.0215) of cysteine and TMT reagent (+229.1629) of lysine and peptide N-termini used as static modifications and oxidation (+15.9949) of methionine used as a variable modification. Reporter ion intensities were reported, with further normalization performed by using the total intensity in each channel to correct discrepancies in sample amount in each channel. The false-discovery rate (FDR) cutoff was 1% for all peptides. Extracted reporter ions were further normalized by using the total intensity in each channel to correct for differences in sample amounts.

### STATISTICAL ANALYSIS

Figures were prepared and statistics were calculated using GraphPad PRISM. Unless otherwise indicated in the figure legends, statistical significance was calculated using an unpaired, two-tailed student’s t-test with 95% confidence intervals. *P < 0.05; **P < 0.01; ***P < 0.001; ****P < 0.0001; NS, not significant (P > 0.05). Statistical details can also be found in the figure legends for each figure.

## Supplementary Material

1 Data S1. Unprocessed source data underlying all blots and graphs. Related to Figures 1–7 and Supplemental Figures 1–7.

2 Data S2. Raw metabolomics data. Related to STAR Methods.

4

## Figures and Tables

**Key resources table:** 

REAGENT or RESOURCE	SOURCE	IDENTIFIER
Antibodies		
UQCRC2	Abcam	Cat# ab14745; RRID: AB2213640
Vinculin	Proteintech	Cat# 26520–1-AP; RRID: AB_2868558
SLC1A3	Cell Signaling Technology	Cat# 4166; RRID: AB_1903991
HPRT1	Santa Cruz Biotechnology	Cat# Sc-376938; RRID: AB_2938532
SLC29A1	Abcam	Cat# ab182023; RRID: AB_2885105
P70 S6 Kinase	Cell Signaling Technology	Cat# 9202S; RRID: AB_331676
Phospho-p70 S6K (Thr389)	Cell Signaling Technology	Cat# 9234S; RRID: AB_2269803
4E-BP1	Cell Signaling Technology	Cat# 9644S; RRID: AB_2097841
Phospho-4E-BP1 (Ser65)	Cell Signaling Technology	Cat# 9451S; RRID: AB_330947
GAPDH	Cell Signaling Technology	Cat# 2118S; mRRID: AB_561053
RPL26	Bethyl Laboratories	Cat# A300–685A-T
Keima	MBL International	Cat# M182–3B; RRID: AB_11142490
LC3B	Sigma Aldrich	Cat# L8918; RRID: AB_1079382
SQSTM1/p62	Cell Signaling Technology	Cat# 88588; RRID: AB_2800125
ATG5	Cell Signaling Technology	Cat# 9980S; RRID: AB_10829153
ATG7	Cell Signaling Technology	Cat# 8558T; RRID: AB_10831194
FLAG	Sigma Aldrich	Cat# F1804; RRID: AB_262044
HSP60	Cell Signaling Technology	Cat# 12165S; RRID: AB_2636980
Anti-Rabbit IgG, HRP-linked	Cell Signaling Technology	Cat# 7074; RRID: AB_2099233
Anti-Mouse IgG, HRP-linked	Cell Signaling Technology	Cat# 7076; RRID: AB_330924
Goat anti-Rabbit IgG Alexa Fluor^™^ 488	Invitrogen	Cat# A-11008
Goat anti-Mouse IgG Alexa Fluor^™^ 555	Invitrogen	Cat# A-21422
Bacterial and virus strains		
LentiCRISPR_v2	Addgene	RRID: Addgene_52961
pMD2.G	Addgene	RRID: Addgene_12259
psPAX2	Addgene	RRID: Addgene_12260
PX458	Addgene	RRID: Addgene_48138
PX458_sgUQCRC2	This paper	N/A
LentiCRISPR_v2_sgHPRT1 #1	This paper	N/A
LentiCRISPR_v2_sgHPRT1 #2	This paper	N/A
LentiCRISPR_v2_sgATG5 #1	This paper	N/A
LentiCRISPR_v2_sgATG5 #2	This paper	N/A
LentiCRISPR_v2_sgATG7 #1	This paper	N/A
LentiCRISPR_v2_sgATG7 #2	This paper	N/A
LentiCRISPR_v2_Scr	This paper	N/A
pLENTI_RPS3_Keima	Addgene	RRID: Addgene_127140
PMXS-NDI1	Addgene	RRID: Addgene_72876
PMXS-Cyto-LbNOX	Kivanc Birsoy’s lab, *Garcia-Bermudez et al*^44^	N/A
PMXS-Mito-LbNOX	Kivanc Birsoy’s lab, *Garcia-Bermudez et al*^44^	N/A
PMXS-SLC1A3	Addgene	RRID: Addgene_72873
PMXS-SLC29A1	This paper	N/A
Biological samples		
Matrigel	Fisher Scientific	CB-40234
		
		
		
		
Chemicals, peptides, and recombinant proteins		
[U-^13^C]glucose	Cambridge Isotope Laboratories	CLM-1396
[U-^13^C]glutamine	Cambridge Isotope Laboratories	CLM-1822
[amide-^15^N]glutamine	Cambridge Isotope Laboratories	NLM-557
[1,2-^13^C]glucose	Cambridge Isotope Laboratories	CLM-504
[^15^N_4_]hypoxanthine	Cambridge Isotope Laboratories	NLM-8500
L-aspartate	Sigma Aldrich	A9256
IACS-010759	ChemieTek	CT-IACS107
(Hydroxypropyl)methyl cellulose	Sigma Aldrich	09963
Dimethyl Sulfoxide (DMSO)	Sigma Aldrich	D1435
RPMI-1640	Sigma Aldrich	R8758
Glutamine-free RPMI	Sigma Aldrich	R0883
Polybrene	Sigma Aldrich	TR-1003-G
Bovine Serum Albumin	Sigma Aldrich	A2153
Dialyzed FBS	Gemini Bio-Products	100108
Lipofectamine 3000 Transfection Reagent	Invitrogen	L3000001
Puromycin	Fisher Scientific	NC9138068
Blasticidin	Fisher Scientific	NC1366670
RIPA buffer	Boston BioProducts	BP-115
Halt^™^ Protease and Phosphatase Inhibitor Cocktail (100X)	Thermo Fisher Scientific	78444
Sodium Azide, Crystalline	Fisher Scientific	S227I-25
Sodium Hydroxide (Pellets/Certified ACS)	Fisher Scientific	S318
Pierce ECL Western Blotting Substrate	Thermo Fisher Scientific	PI32106
Acetonitrile, Optima LC/MS Grade	Fisher Scientific	A955–4
Methanol, Optima LC/MS Grade	Fisher Scientific	A456–4
Water, Optima LC/MS Grade	Fisher Scientific	W64
Formic Acid, 99.0+%, Optima LC/MS Grade	Fisher Scientific	A117–50
Ammonium bicarbonate	Sigma Aldrich	A6141
Pyridine	Sigma Aldrich	270407
N-(tert-butyldimethylsilyl)-N-methyltrifluoroacetamide (MTBSTFA)	Sigma Aldrich	394882
Hoechst	Thermo Fisher Scientific	62249
Propidium Iodide (PI)	Thermo Fisher Scientific	P3566
Pyruvate	Sigma Aldrich	S8636
Uridine	Sigma Aldrich	U3003
Sodium 2-oxobutyrate (AKB)	Millipore Sigma	K0875
Inosine	Millipore Sigma	I4125
Guanosine	Millipore Sigma	G6752
Adenosine	Millipore Sigma	A9251
NBMPR	Millipore Sigma	N2255
Nocodazole	Fisher Scientific	50–194-7956
Lometrexol	Sigma Aldrich	SML0040
Methotrexate	Selleck Chemicals	S1210
Ketamine/xylazine	ARC Drug Services	N/A
Fibronectin	Sigma Aldrich	F1141
Paraformaldehyde	Electron Microscopy Sciences	15710
Profade-Antifade	Invitrogen	P36935
Seahorse medium	Agilent Technologies	102353
L-glutamine	Thermo Fisher Scientific	G7513
Penicillin-Streptomycin	Millipore Sigma	P0781
D-(+)-Glucose	Honeywell	G8270
Oligomycin A	Sigma Aldrich	O4876
Carbonyl cyanide m-chlorophenylhydrazone (CCCP)	Sigma Aldrich	C2759–10
Rotenone	Sigma Aldrich	R8875
Antimycin	Sigma Aldrich	A8674
Trizol	Thermo Fisher Scientific	15596018
Tirethylammonium bicarbonate (TEAB)	Fisher Scientific	90114
Critical commercial assays		
Precise HPRT1 assay Kit	Novo CIB	K0709–01-2
*DC*^™^ Protein Assay Kit II	BIO-RAD	5000112
Pierce BCA protein assay Kit	Thermo Scientific	23225
RNeasy Mini Kit	Qiagen	74106
Qubit RNA High Sensitivity Kit	Invitrogen	Q32852
NEBNext Ultra II Directional RNA Library Prep Kit for Illumina	New England Biolabs	E7490, E7760
NEBNext^®^ Multiplex Oligos for Illumina	New England Biolabs	E7730L, E7735L, E7500L
		
Deposited data		
RNA-seq data	This paper	GSE265923
Proteomics data	This paper	Mass IVE (MSV000094553)
Data values to generate graphs in the paper	This paper	Data S1
Metabolomics data	This paper	Data S2
Experimental models: Cell lines		
Human: H460	Hamon Cancer Center Collection at the University of Texas Southwestern Medical Center	N/A
Human: 293T	American Type Culture Collection (ATCC)	CRL-3216
Human: 786-O	American Type Culture Collection (ATCC)	CRL-1932
Human: H157	Hamon Cancer Center Collection at the University of Texas Southwestern Medical Center	N/A
Human: A549	American Type Culture Collection (ATCC)	CCL-185
Human: SK-N-DZ	American Type Culture Collection (ATCC)	CRL-2149
Experimental models: Organisms/strains		
Mouse: NOD.CB17-Prkdc^*scid*^Il2rg^*tm1Wjl*^/ SzJ (NSG)	The Jackson Laboratory	N/A
		
		
		
		
		
		
		
		
Oligonucleotides		
sgUQCRC2	Integrated DNA Technologies (IDT)	GCAAAGGCCAACTACCGTGG
sgHPRT1 #1	Integrated DNA Technologies (IDT)	TTATGGCGACCCGCAGCCC
sgHPRT1 #2	Integrated DNA Technologies (IDT)	TCTTGCTCGAGATGTGATGA
sgATG5 #1	Integrated DNA Technologies (IDT)	TGATATAGCGTGAAACAAGT
sgATG5 #2	Integrated DNA Technologies (IDT)	ATCACAAGCAACTCTGGAT
sgATG7 #1	Integrated DNA Technologies (IDT)	CCCGTTGCTGCCCAGCTAT
sgATG7 #2	Integrated DNA Technologies (IDT)	TCCAAGGCACTACTAAAAG
sgScr	Integrated DNA Technologies (IDT)	TTCTTAGAAGTTGCTCCACG
		
		
Software and algorithms		
Prism Graphpad	Graphpad Software	https://www.graphpad.com/
R Studio	Posit Software	N/A
Fiji	NIH	https://imagej.net/software/fiji/downloads
TraceFinder^™^	Thermo Scientific	N/A
MultiQuant version 2.1	Applied Biosystems SCIEX	N/A
MassHunter Profinder	Agilent Technologies	N/A
Proteome Discoverer V.3.0 SP1	Thermo Scientific	N/A
Other		
MetaboAnalyst 5.0	Pang, Z. et al^70^.	doi:10.1093/nar/gkab382
		
		
		
		

## Highlights

- ETC inhibition rewires purine metabolism
- Cytosolic NAD(H) imbalance induces purine accumulation in ETC-deficient cells
- HPRT1-mediated purine salvage supports NSCLC growth during ETC inhibition
- Purine uptake is required to supply salvage upon ETC inhibition
