# Supplementary material for: Electron transport chain inhibition increases cellular dependence on purine transport and salvage
Source: Cell Metab. Author manuscript; Available in PMC 2024 Jul 12. (PMC11240302; doi:10.1016/j.cmet.2024.05.014)

Figure 2K

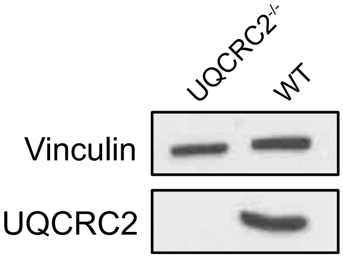
UQCRC2-/-
WT
Vinculin
UQCRC2

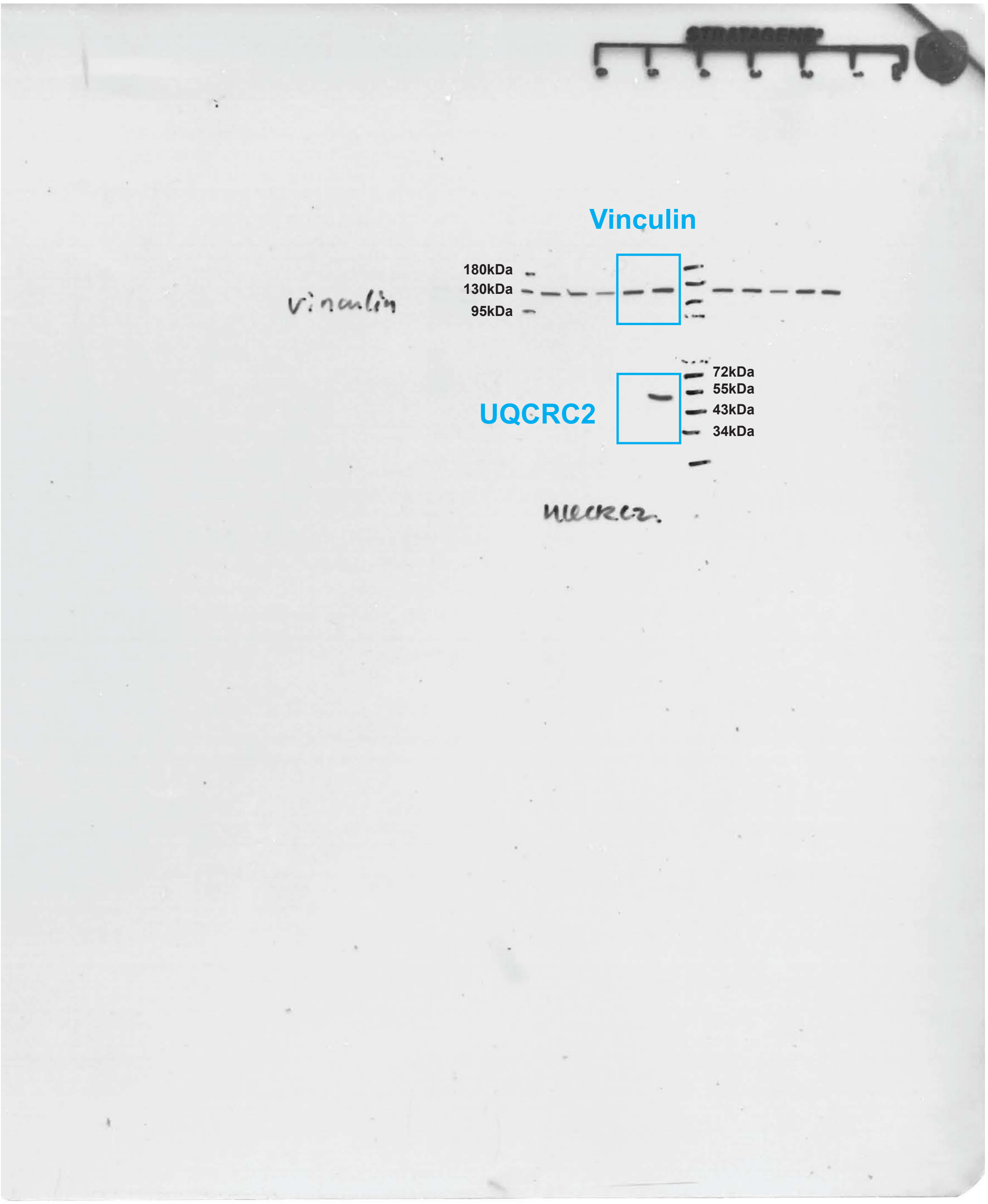
Vinculin
180kDa
130kDa
95kDa
UQCRC2
72kDa
55kDa
43kDa
34kDa

Figure 3B

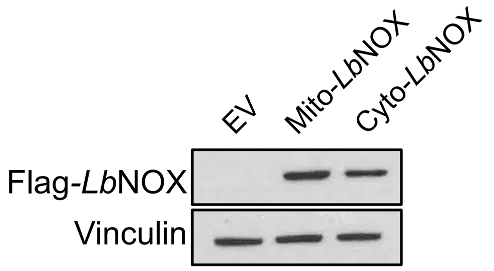

EV
Mito-*Lb*NOX
Cyto-*Lb*NOX
Flag-*Lb*NOX
Vinculin

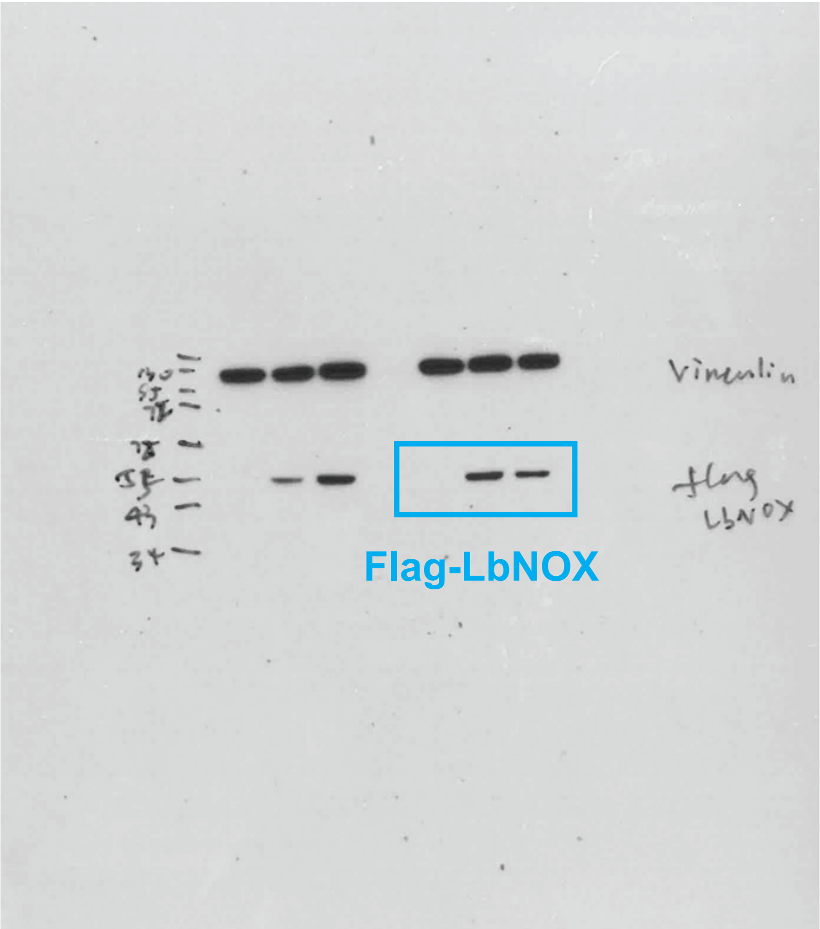

Vinculin
Flag-LbNOX

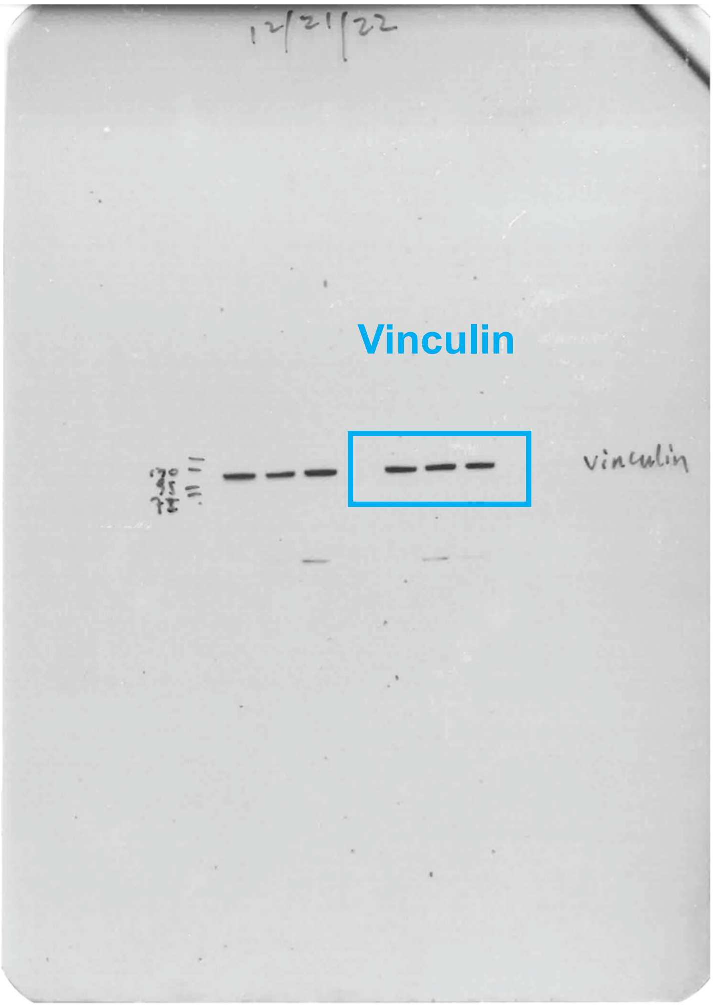

12/21/22
Vinculin
vinculin

Figure 4I

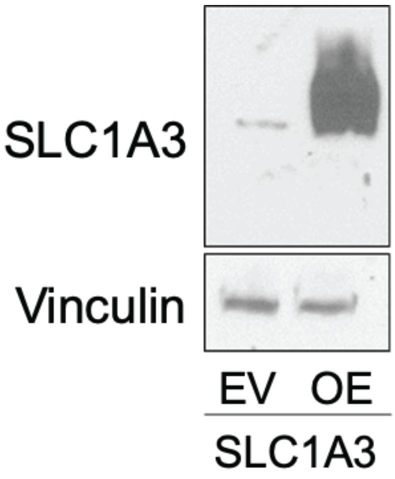

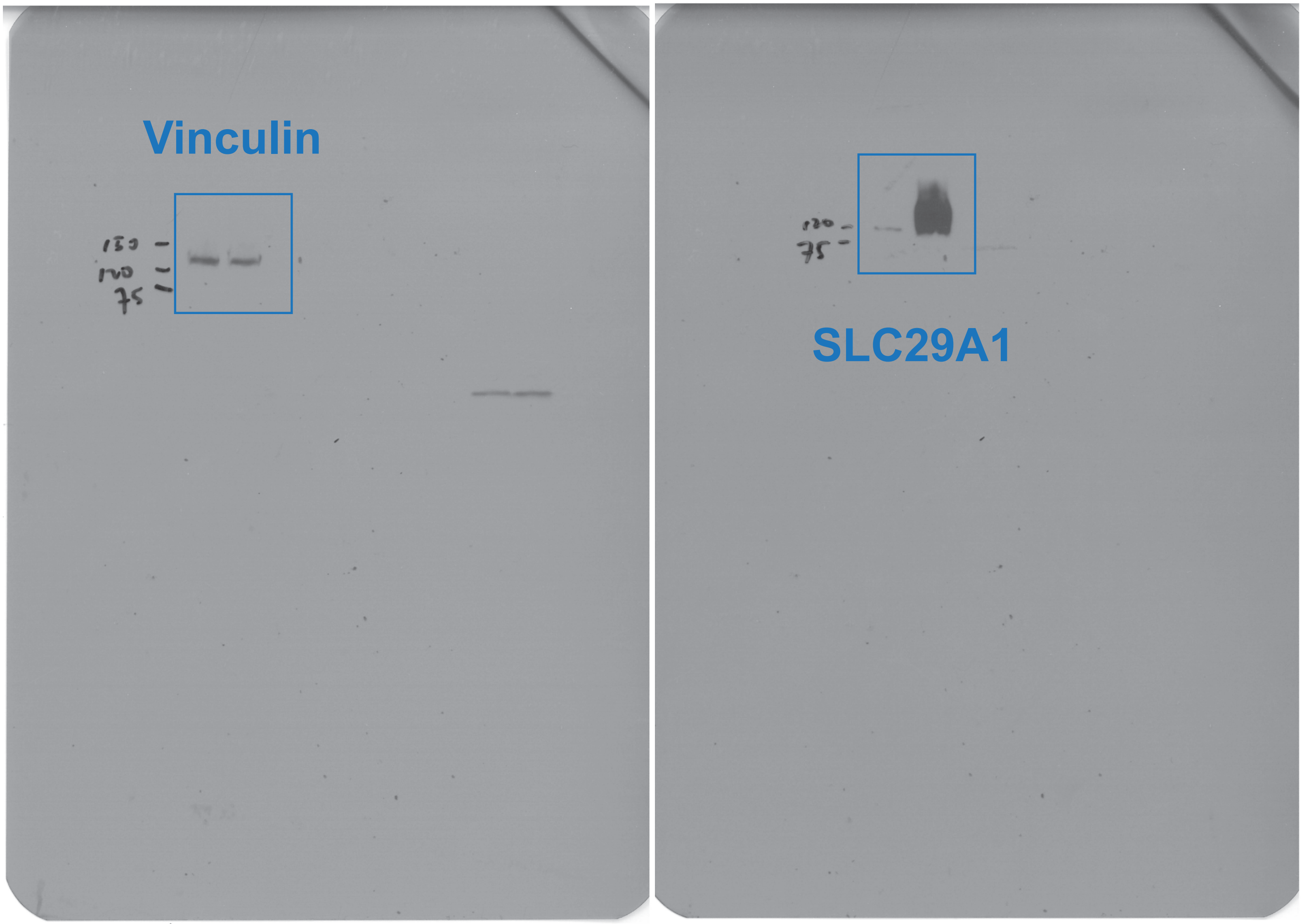

Figure 7E

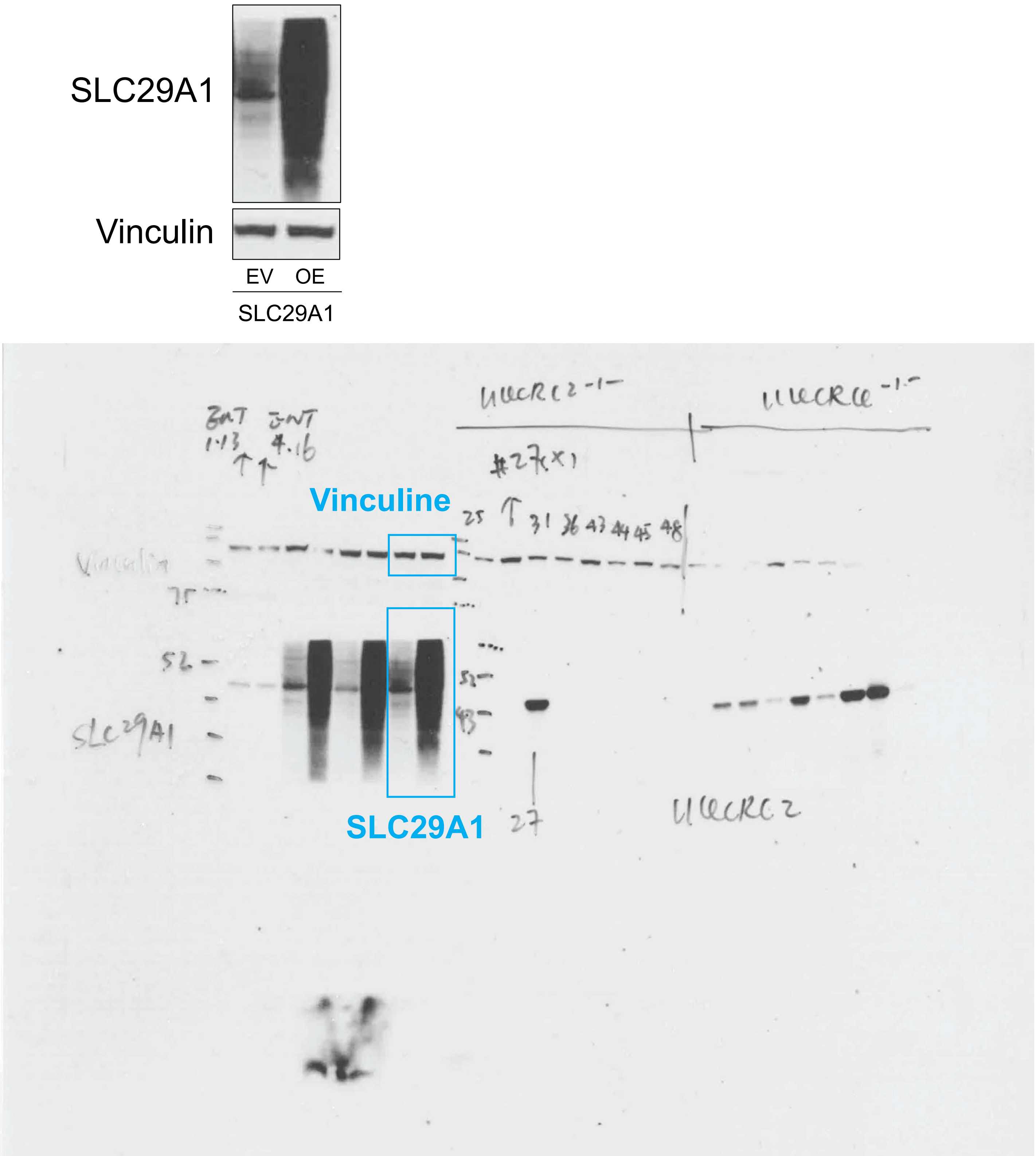

Supplemental Figure 5D

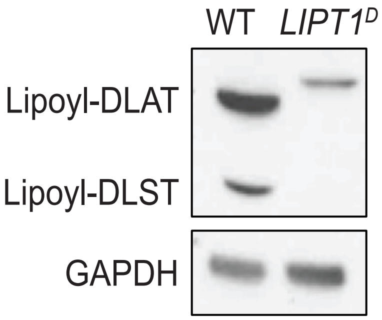

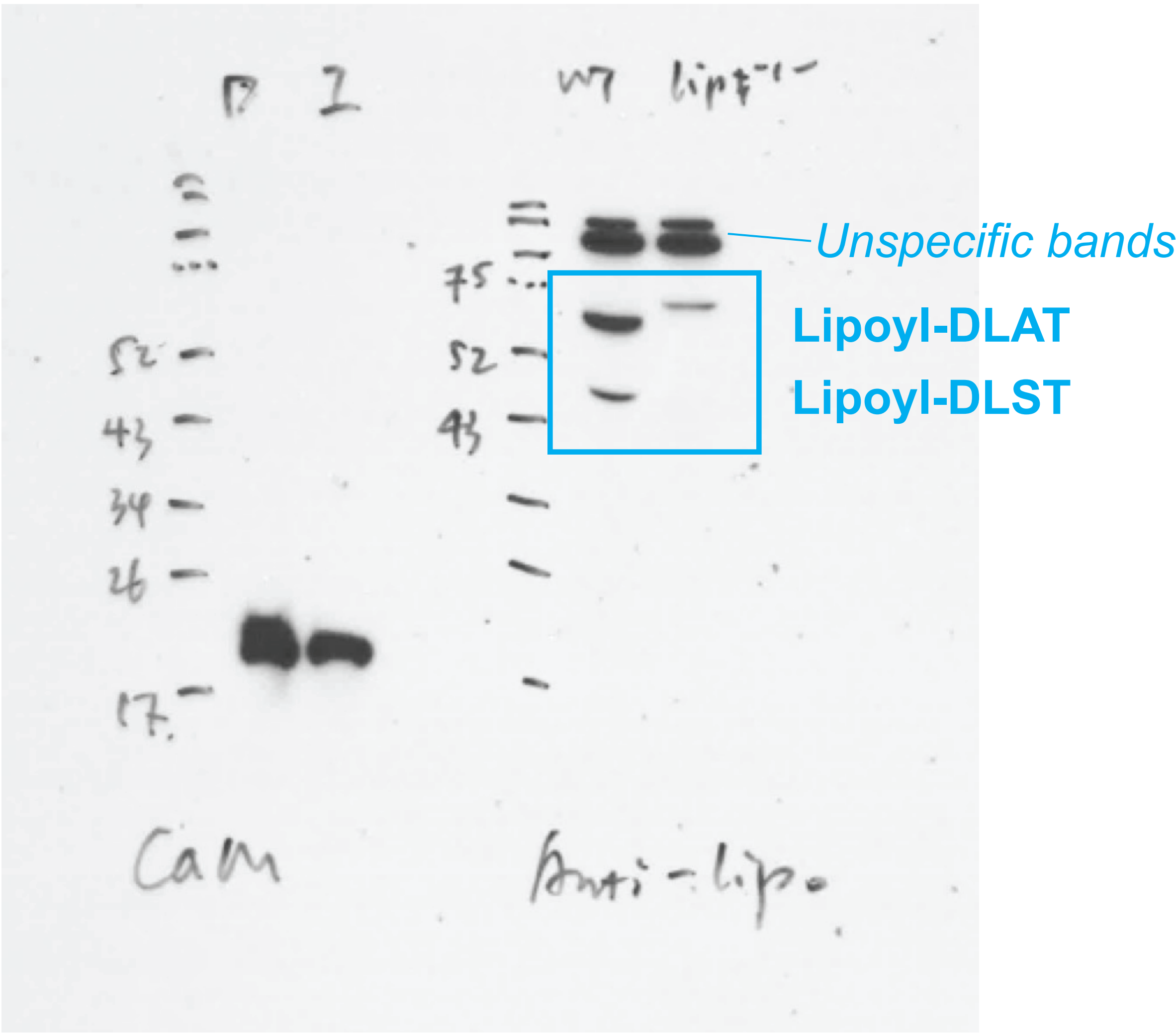

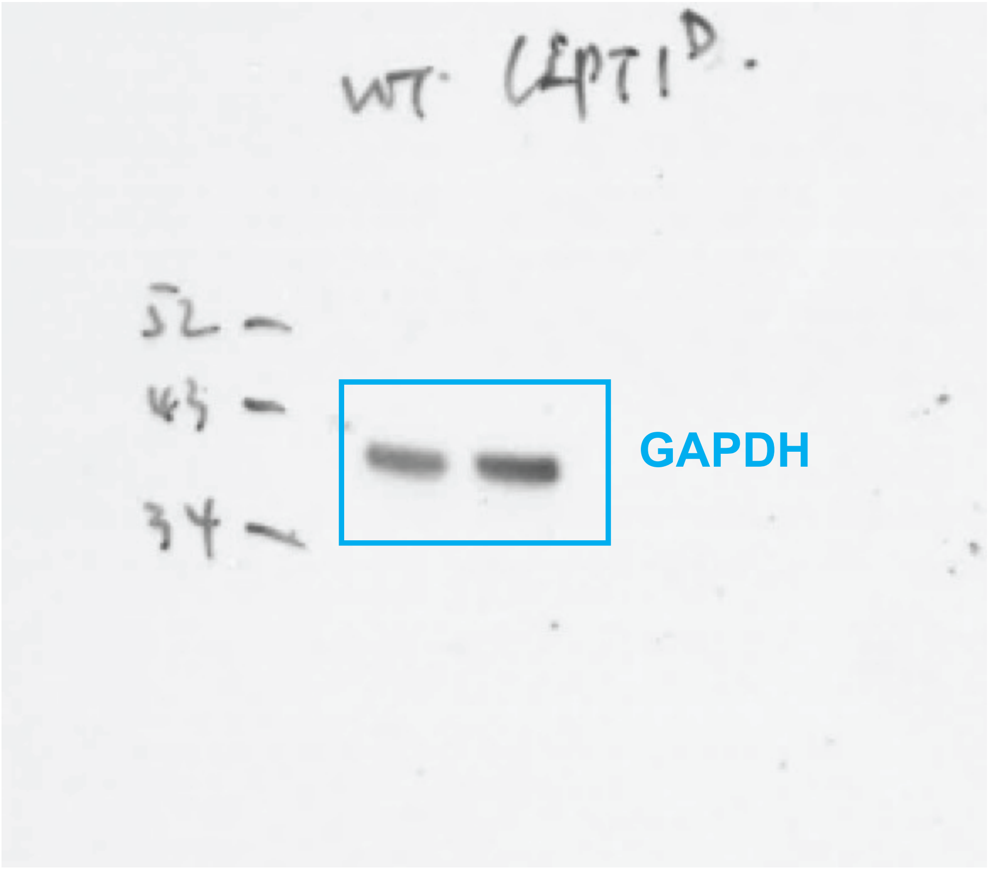

Supplemental Figure 5F

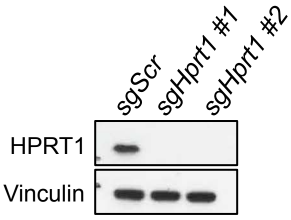

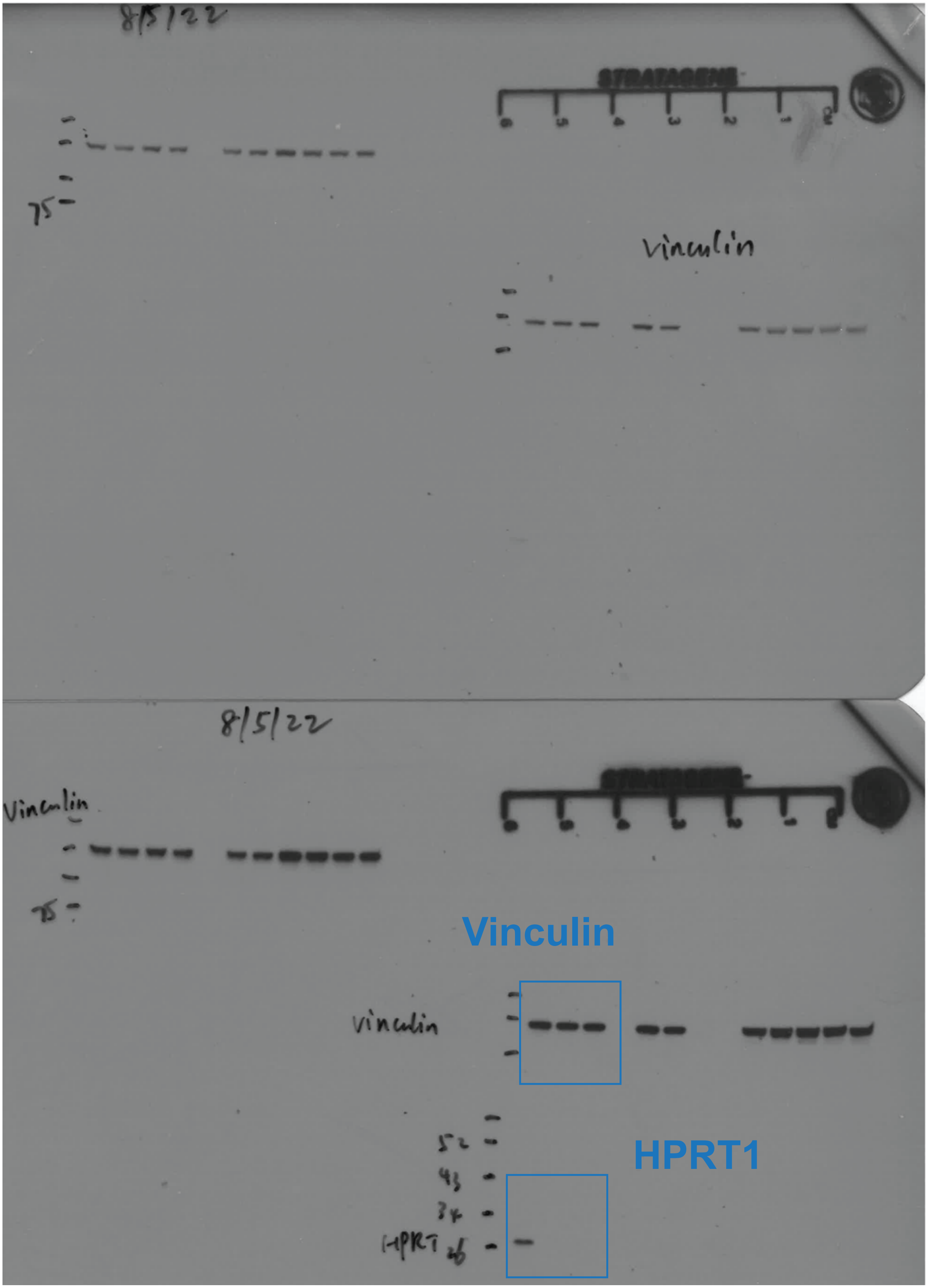

Supplemental Figure 7A - Part I

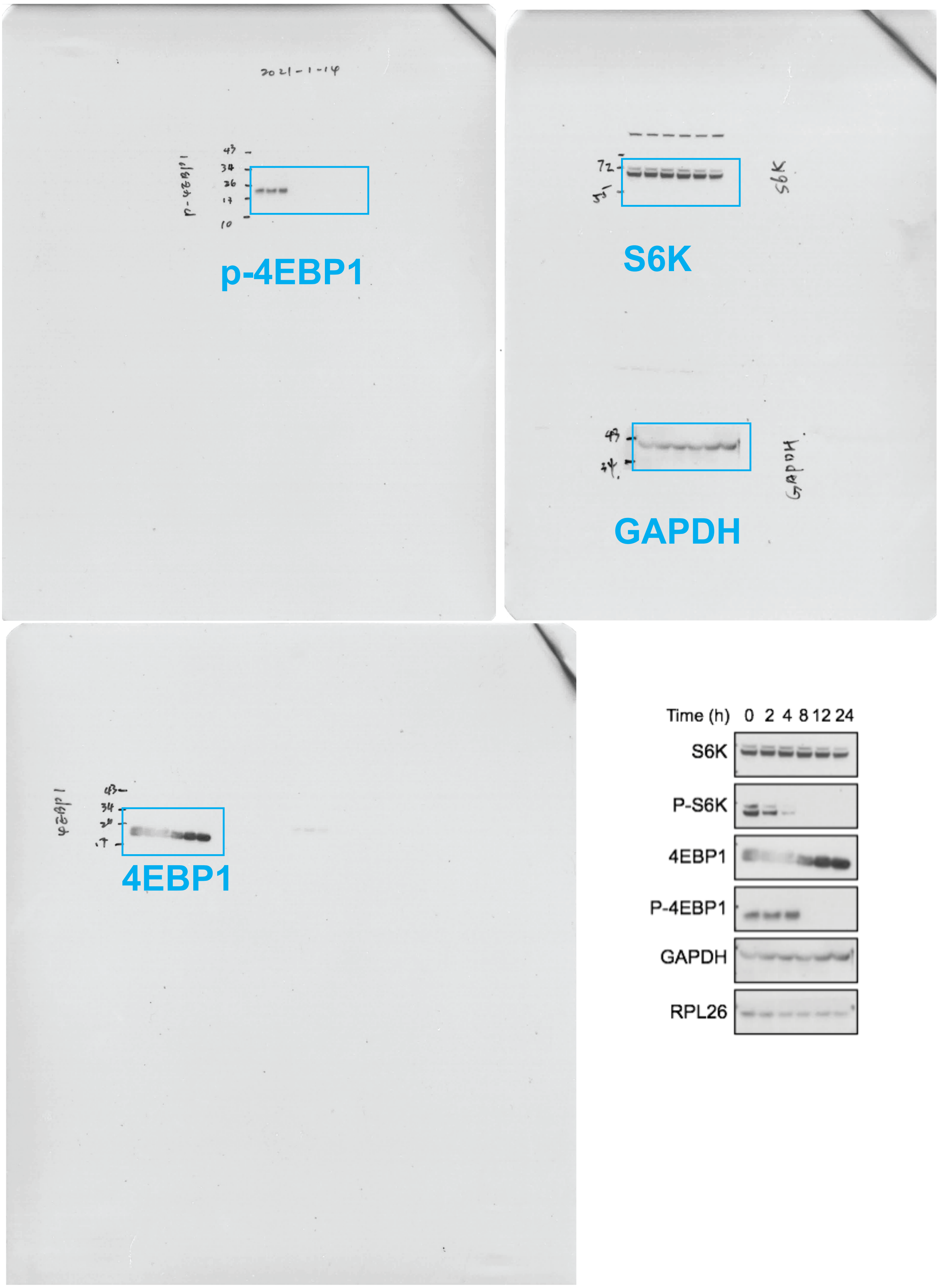

Supplemental Figure 7A - Part II

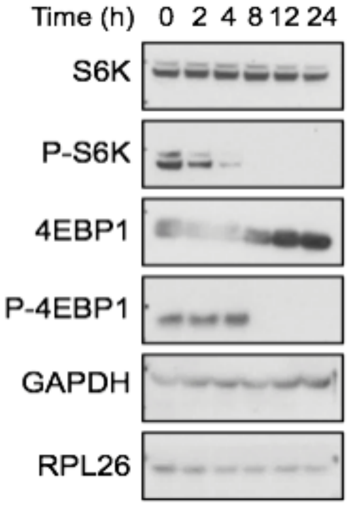

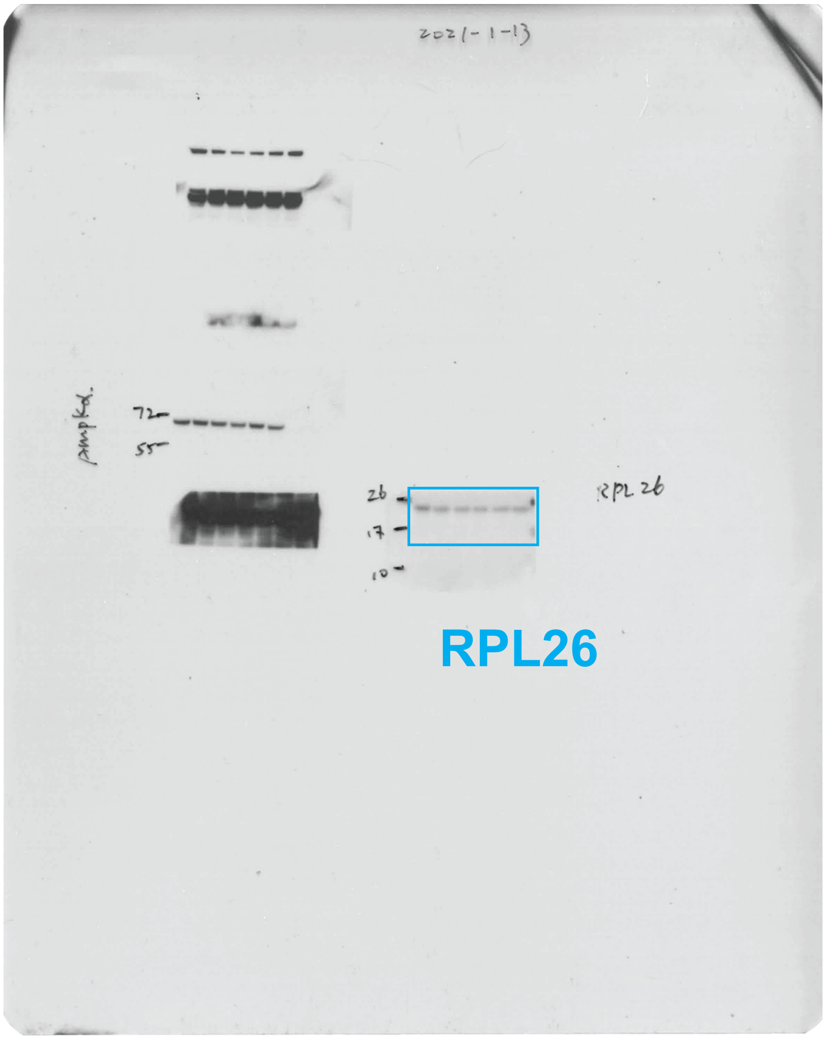

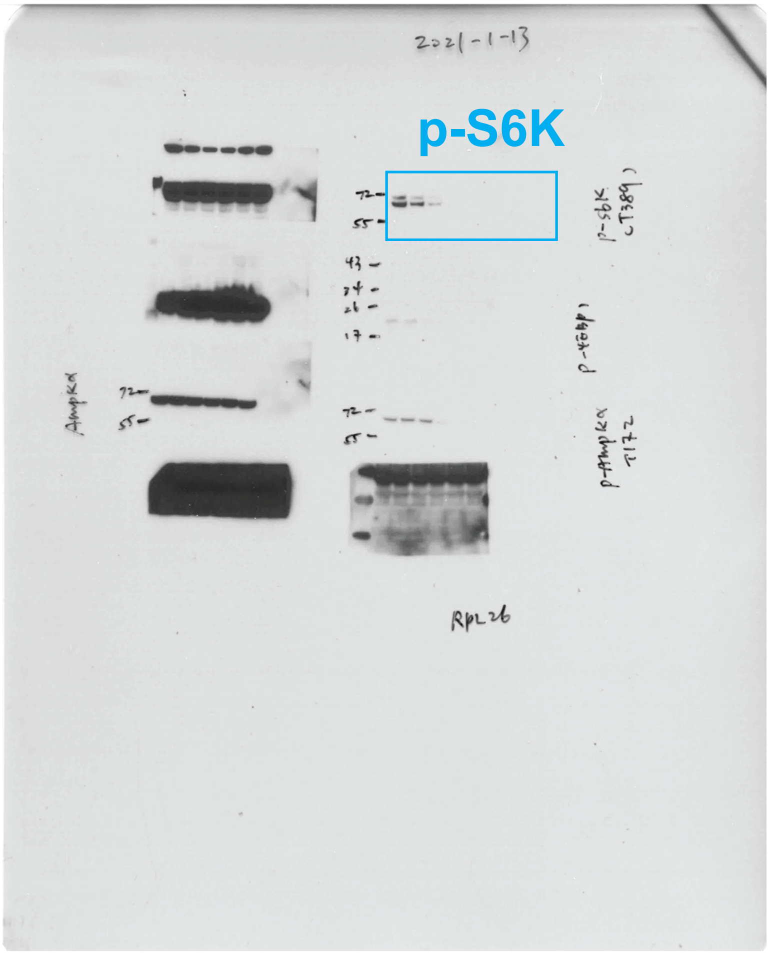

Supplemental Figure 7B

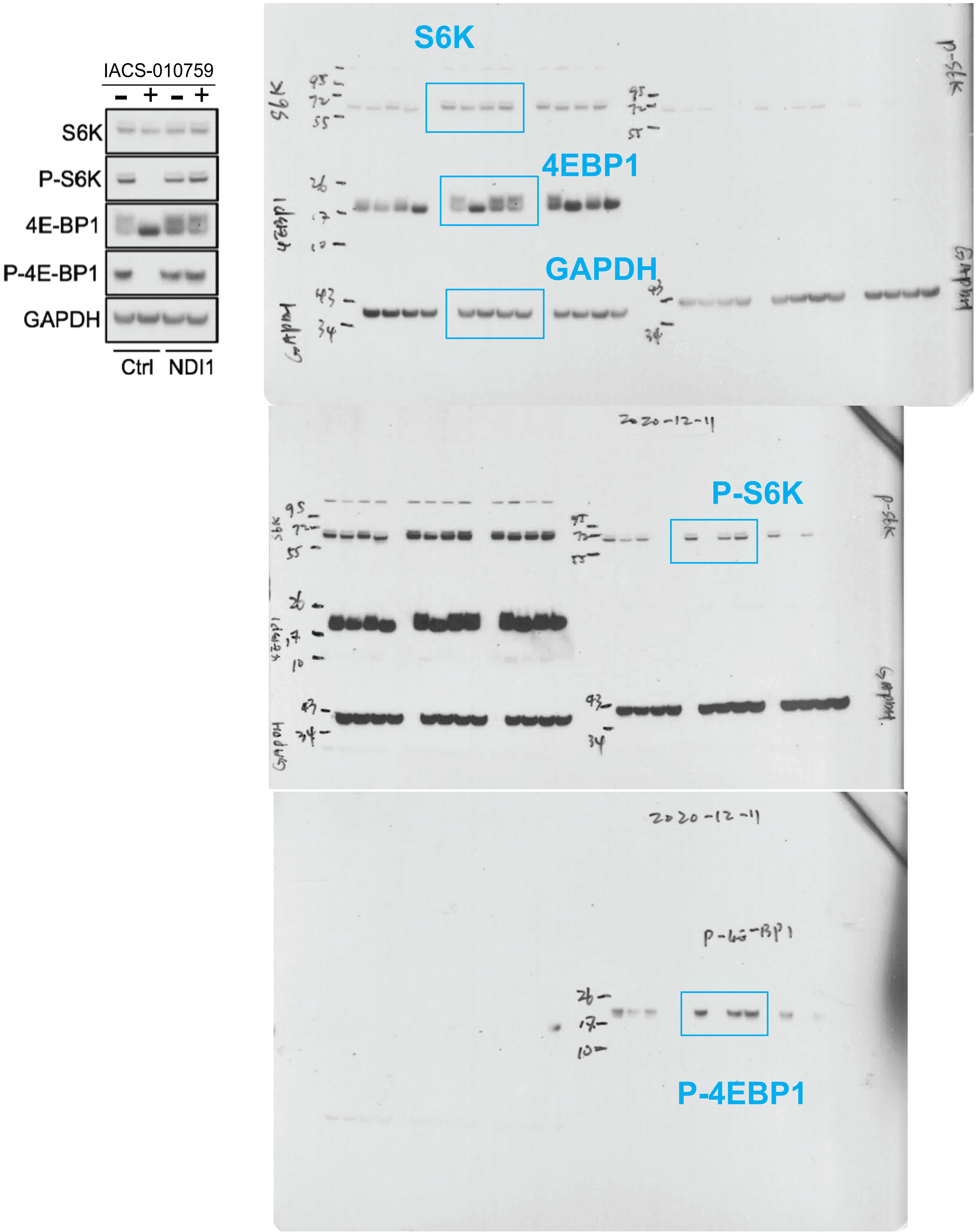

Supplemental Figure 7C

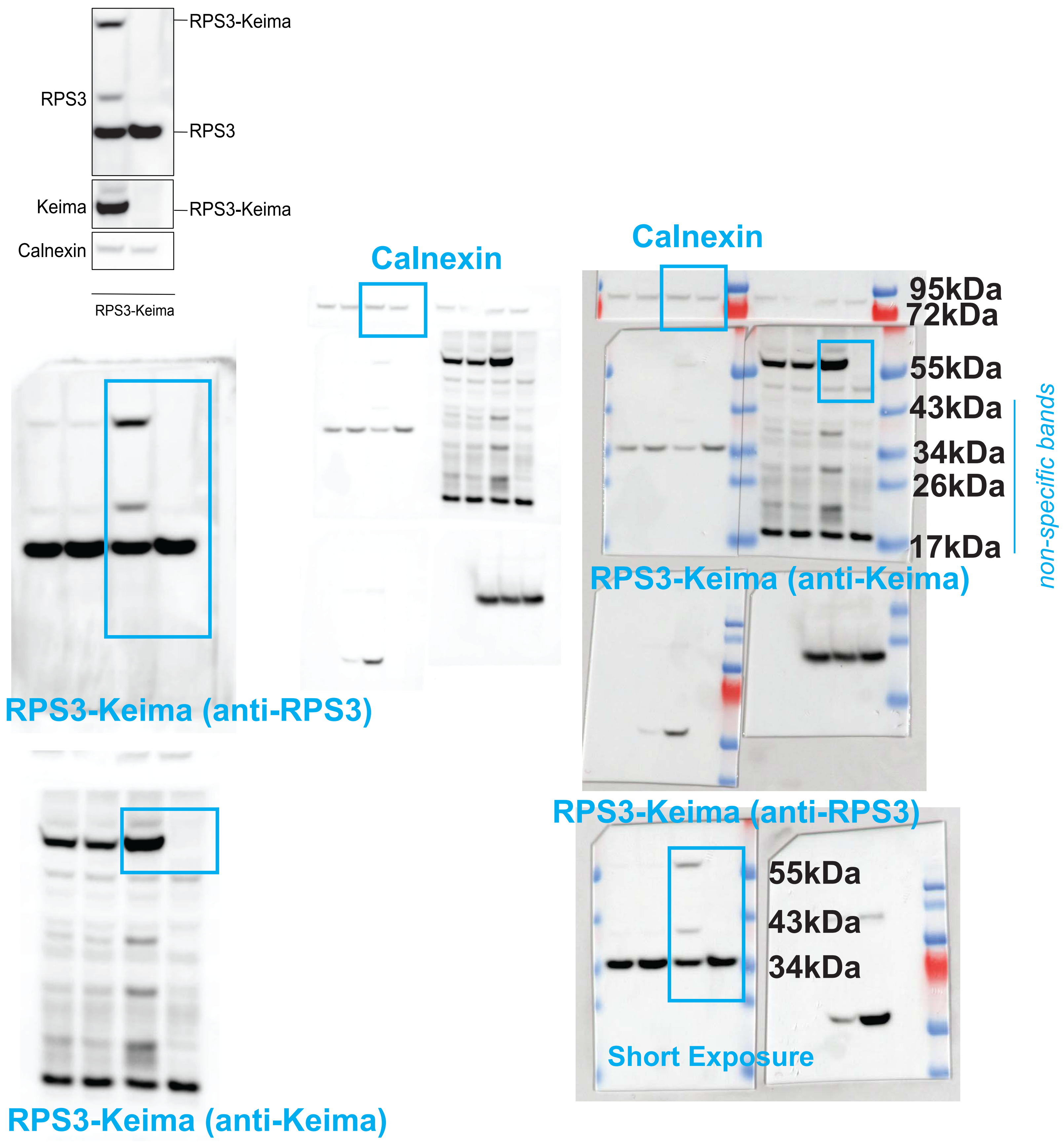

Supplemental Figure 7D

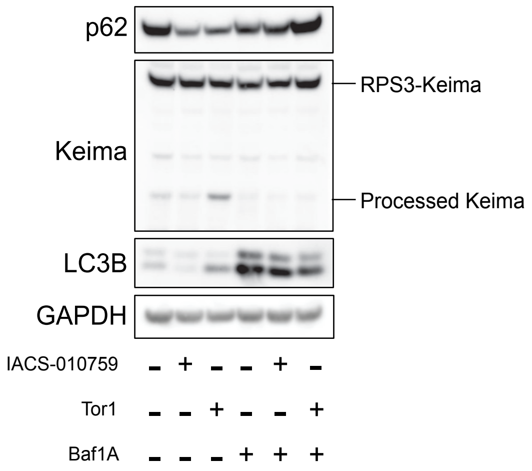

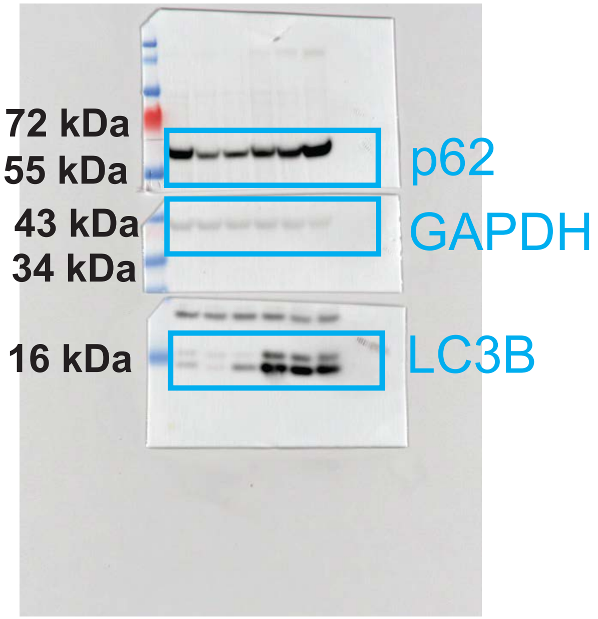

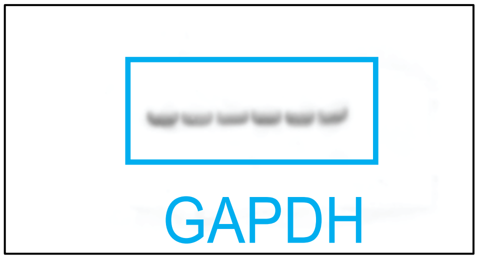

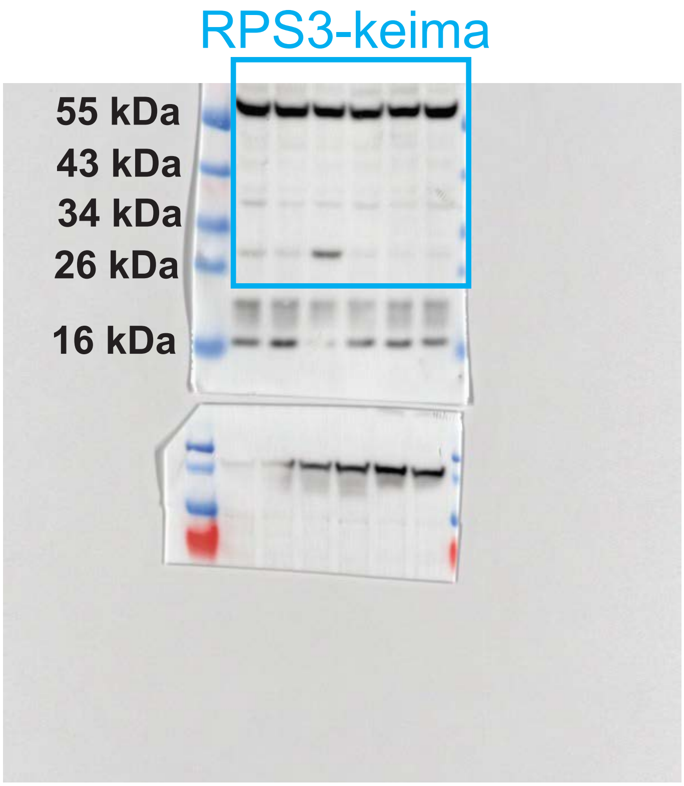

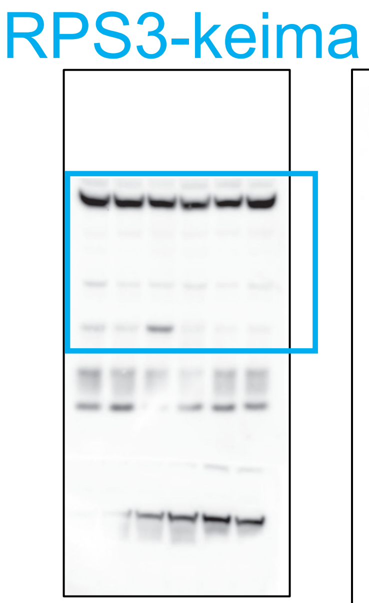

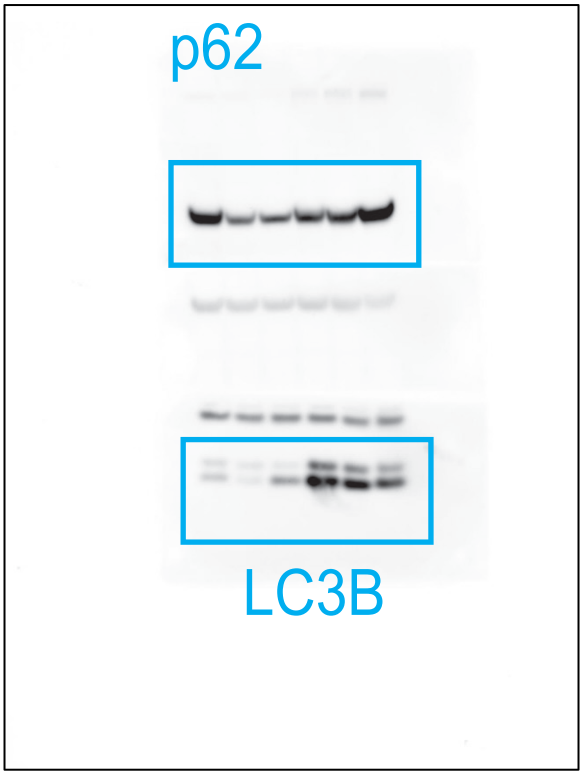

Supplemental Figure 7E

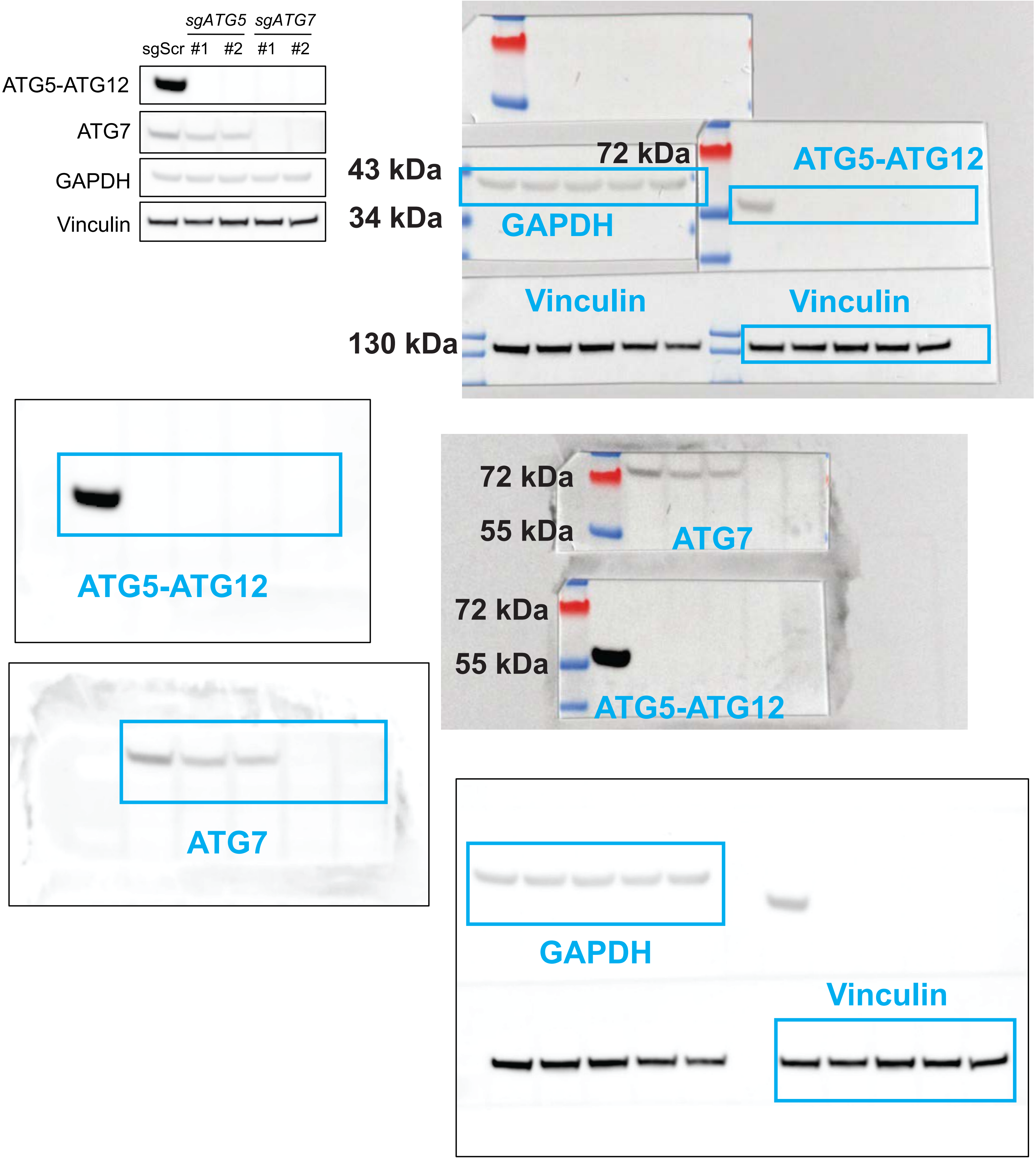

Supplement: 1 — Data S1. Unprocessed source data underlying all blots and graphs. Related to Figures 1–7 and Supplemental Figures 1–7. [file NIHMS2004245-supplement-1.zip › Uncropped Western blot figures.pdf]
