# Supplementary material for: Electron transport chain inhibition increases cellular dependence on purine transport and salvage
Source: Cell Metab. Author manuscript; Available in PMC 2024 Jul 12. (PMC11240302; doi:10.1016/j.cmet.2024.05.014)
Supplement: 4 [file NIHMS2004245-supplement-4.pdf]

Supplemental Figure 1 (Related to Figure 2)

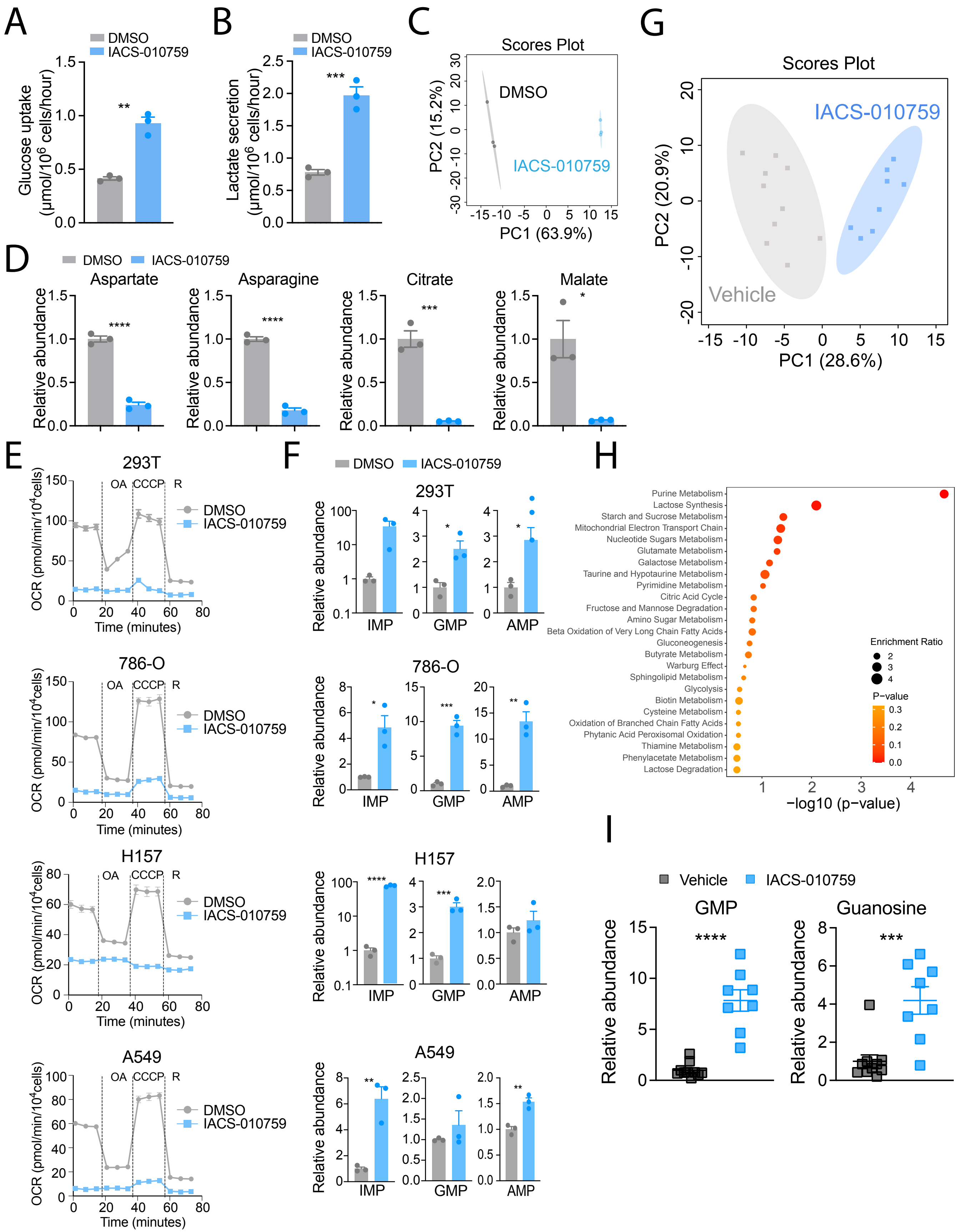

Figure S1. ETC complex I blockade induces purine nucleotide accumulation, related to Figure 2.

A-B. Glucose uptake (**A**) and lactate secretion (**B**) in H460 cells treated with DMSO or 25 nM IACS-010759 for 6 hours (n=3).

C. Principal component analysis of metabolomic profiles in H460 cells treated with DMSO or 25 nM IACS-010759 for 24 hours.

D. Relative abundance of the indicated metabolites in H460 cells treated with DMSO or 25 nM IACS-010759 (n=3).

E. Oxygen consumption rates (OCR) in the indicated cell lines pre-treated with DMSO or 25 nM IACS-010759. OA: oligomycin A; CCCP: Carbonyl cyanide *m*-chlorophenylhydrazone; R: rotenone. Data are from one of three independent experiments.

F. Relative purine nucleotide abundance in the indicated cell lines treated with DMSO or 25 nM IACS-010759 for 24 hours (n=3).

G. Principal component analysis of metabolomic profiles in H460 xenografts treated with vehicle or IACS-010759 for five days.

H. Metabolite set enrichment analysis comparing vehicle and IACS-010759-treated H460 xenografts.

I. Relative abundance of the indicated metabolites in H460 xenografts treated with vehicle or IACS-010759 for five days. Vehicle (n=10), IACS-010759 (n=8).

Unpaired, two-sided t tests were used for the statistical analyses. ****: $P < 0.0001$; ***: $P < 0.001$; **: $P < 0.01$, *: $P < 0.05$. Error bars denote SEM.

Supplemental Figure 2 (Related to Figure 2)

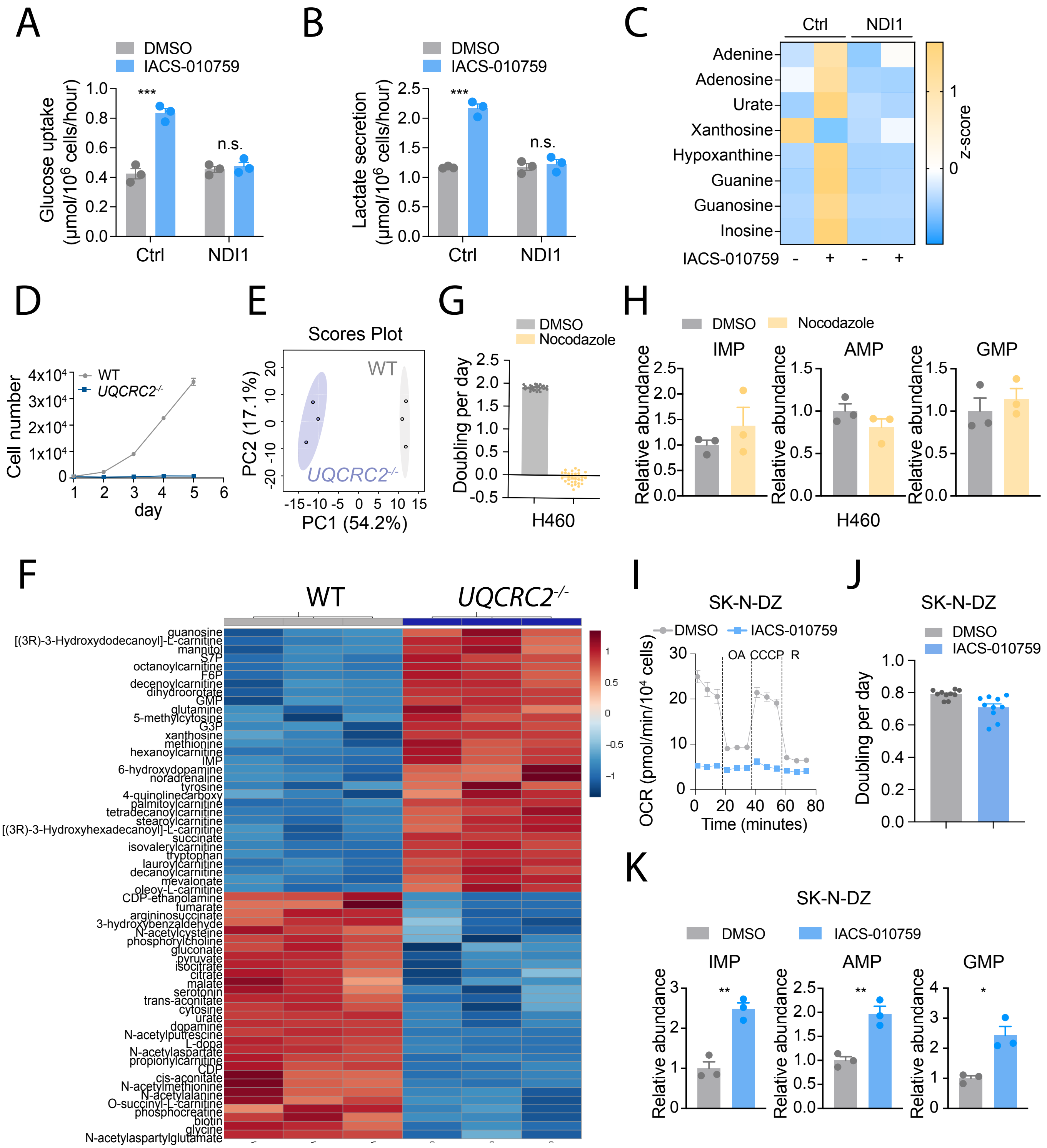

Figure S2. ETC deficiency induces purine nucleotide accumulation independently of cell growth arrest, related to Figure 2.

A-B. Glucose uptake (**A**) and lactate secretion (**B**) in H460 cells treated with DMSO or 25 nM IACS-010759 for 6 hours (n=3).

C. Heatmap showing purine metabolite abundance in control and NDI1-expressing cells treated with DMSO or 25 nM IACS-010759 for 24 hours.

D. Growth of WT and *UQCRC2*$^{-/-}$ H460 cells. Data are from one of three independent experiments.

E. Principal component analysis of metabolomic profiles in WT and *UQCRC2*$^{-/-}$ H460 cells.

F. Heatmap showing metabolomic profiles in WT and *UQCRC2*$^{-/-}$ H460 cells.

G. Growth rates of H460 cells treated with DMSO or 100 nM nocodazole. Data are from one of three independent experiments.

H. Relative purine nucleotide abundance in the indicated cell lines treated with DMSO or 100 nM nocodazole for 24 hours (n=3).

I. OCR in SK-N-DZ cells pre-treated with DMSO or 25 nM IACS-010759 for 24 hours. Data are from one of three independent experiments.

J. Growth rates of SK-N-DZ cells treated with DMSO or 25 nM IACS-010759. Data are one of three independent experiments.

K. Relative purine nucleotide abundance in SK-N-DZ cells treated with DMSO or 25 nM IACS-010759 for 24 hours (n=3).

Unpaired, two-sided t tests were used for the statistical analyses. **: $P < 0.01$; *: $P < 0.05$. n.s.: $P > 0.05$. Error bars denote SEM.

Supplemental Figure 3 (Related to Figure 3)

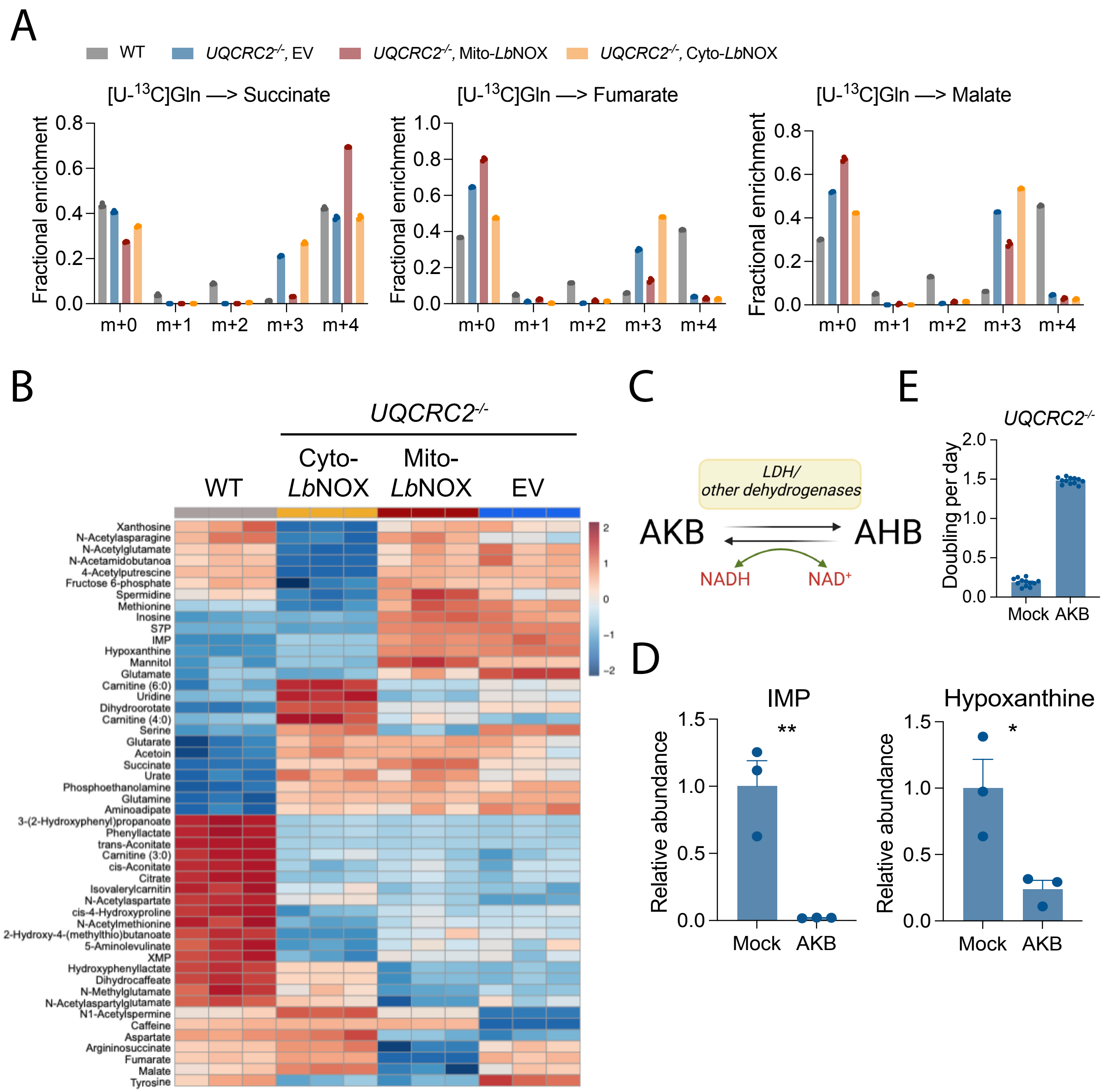

Figure S3. Effects of compartmentalized redox manipulation on metabolism during ETC blockade, related to Figure 3.

A. Isotopologue fractions in succinate, fumarate, and malate after 6 hours of culture with [U-^{13}C]glutamine in WT and *UQCRC2*$^{-/-}$ cells expressing empty vector (EV), Mito-*Lb*NOX or Cyto-*Lb*NOX (n=3).

B. Heatmap showing metabolomic profiles in WT and *UQCRC2*$^{-/-}$ H460 cells that express empty vector (EV), Mito-*Lb*NOX or Cyto-*Lb*NOX.

C. Schematic illustrating how AKB mitigates cytosolic NADH reductive stress.

D. Relative abundance of IMP and hypoxanthine in *UQCRC2*$^{-/-}$ cells cultured with or without 1 mM AKB (n=3).

E. Growth rates of *UQCRC2*$^{-/-}$ cells cultured with or without 1 mM AKB (n=12). Data are from one of three independent experiments.

**: P < 0.01; *: P < 0.05. Error bars denote SEM. BioRender was used to generate the illustration.

Supplemental Figure 4 (Related to Figure 4)

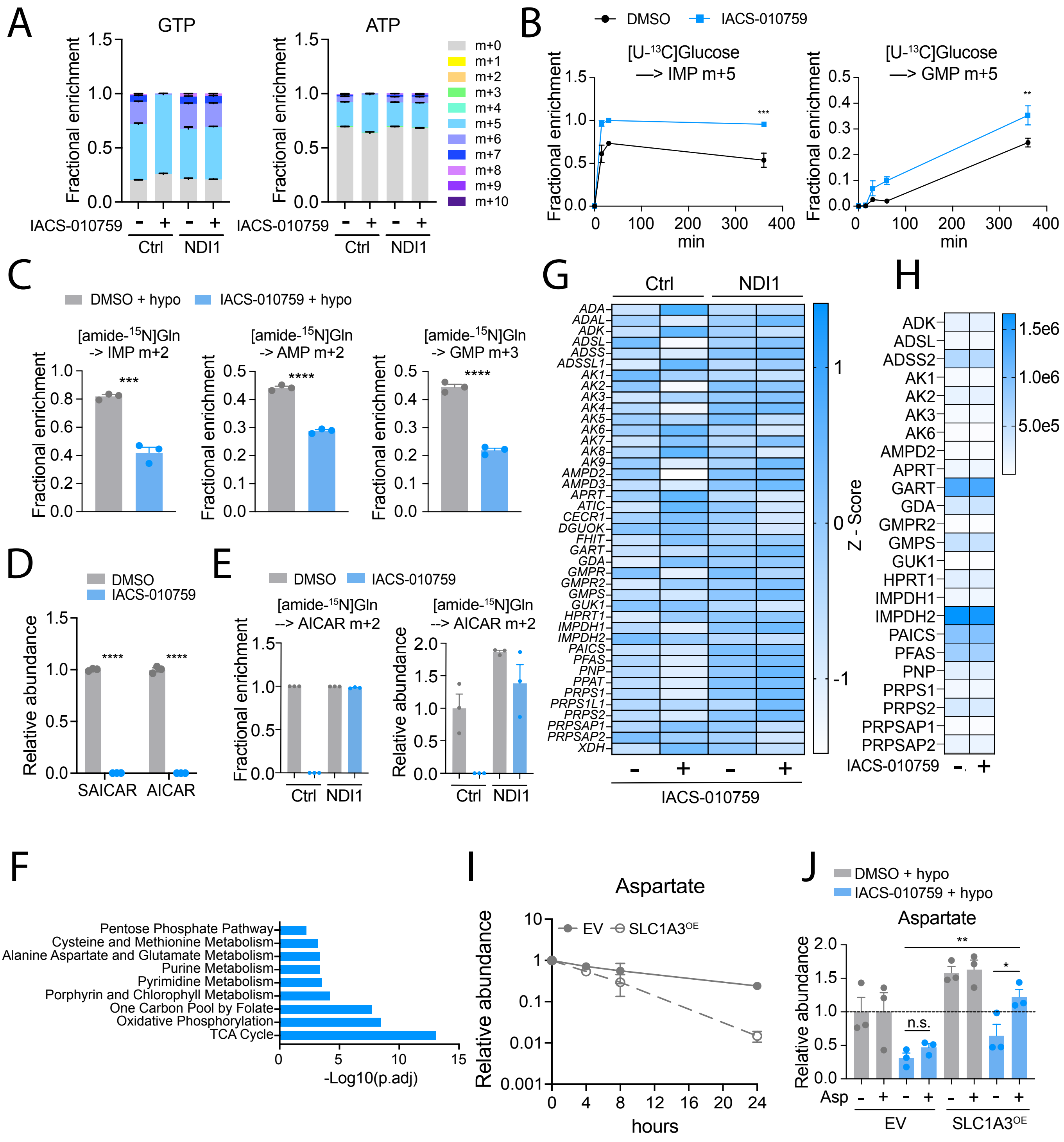

Figure S4. Functional OXPHOS is important for de novo purine nucleotide synthesis, related to Figure 4.

A. Isotopologue fractions in GTP and ATP after 6 hours of culture with [U-^{13}C]glucose in control and NDI1-expressing H460 cells treated with DMSO or 25 nM IACS-010759 for 24 hours (n=3).

B. Time-dependent labeling of m+5 IMP and m+5 GMP in H460 cells pre-treated with DMSO or 25 nM IACS-010759 for 24 hours prior to culture with [U-^{13}C]glucose (n=3 at each time point).

C. Fractional enrichment of m+2 IMP, m+2 AMP, and m+3 GMP after 6 hours of culture with [amide-^{15}N]glutamine and 10 µM hypoxanthine (hypo), following 24-hours of treatment with DMSO or 25 nM IACS-010759 (n=3).

D. Relative abundance of SAICAR and AICAR in H460 cells treated with DMSO or 25 nM IACS-010759 for 24 hours (n=3).

E. Fractional enrichment and relative abundance of m+2 AICAR after 5 minutes of culture with [U-^{13}C]glucose in control and NDI1-expressing H460 cells pre-treated with DMSO or 25 nM IACS-010759 for 24 hours (n=3).

F. Pathway analysis using genes that share co-essentiality with *PPAT*.

G. Heatmap showing mRNA levels (FPKM) of purine pathway-associated genes in control and NDI1-expressing H460 cells treated with DMSO or 25 nM IACS-010759 for 24 hours.

H. Heatmap showing protein abundance of enzymes from the purine metabolic pathway in H460 cells treated with DMSO or 25 nM IACS-010759 for 24 hours.

I. Relative abundance of aspartate in aspartate-supplemented media during culture of empty vector-expressing control cells (EV) and SLC1A3-overexpressing (SLC1A3^{OE}) cells.

J. Relative abundance of aspartate in control (EV) and SLC1A3^{OE} cells pretreated with or without DMSO, 25 nM IACS-010759, and 150 µM aspartate (Asp) after 6 hours of culture with [amide-^{15}N]glutamine and 10 µM hypoxanthine (hypo) (n=3).

Unpaired, two-sided t tests were used for the statistical analyses. ****: $P < 0.0001$; ***: $P < 0.001$; **: $P < 0.01$; *: $P < 0.05$; n.s.: $P > 0.05$. Error bars denote SEM.

Supplemental Figure 5 (Related to Figure 5)

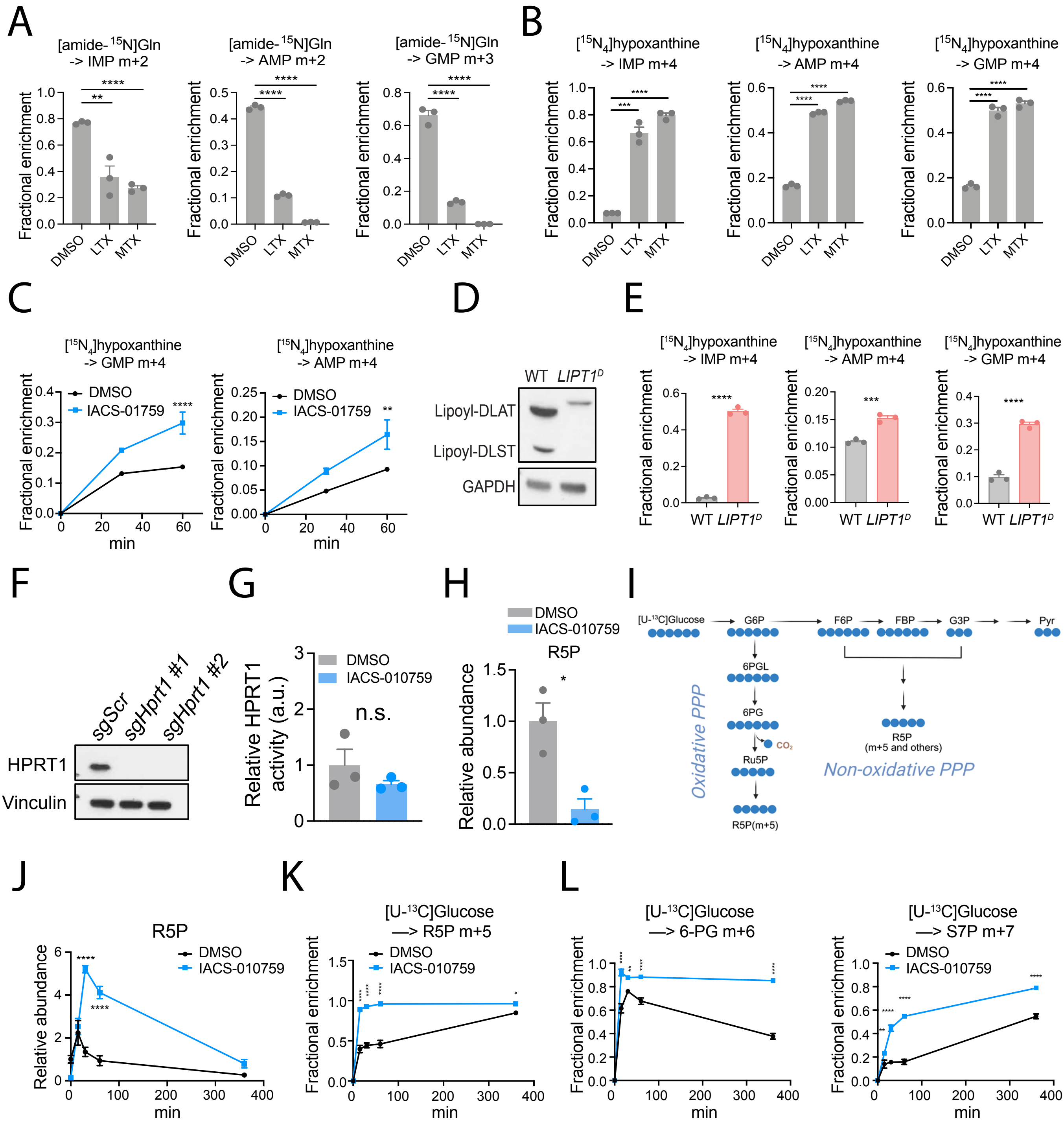

Figure S5. ETC deficiency enhances purine salvage and PPP, related to Figure 5.

A-B. Fractional enrichment of m+2 IMP, m+2 AMP, m+3 GMP (**A**) and m+4 IMP, m+4 AMP, and m+4 GMP (**B**) in H460 cells after 6 hours of culture with [amide-^{15}N]glutamine, following 24-hours of treatment with DMSO, 5 µM LTX, or 5µM MTX (n=3).

C. Time-dependent m+4 enrichment in GMP and AMP during culture with [$^{15}N_4$]hypoxanthine in H460 cells pre-treated with DMSO or 25 nM IACS-010759 for 24 hours (n=3).

D. Western blot showing defective lipoylation in LIPT1-deficient (*LITP1^D*) cells. The blot was probed with an antibody that recognizes lipoylated dihydrolipoamide S-Acetyltransferase (DLAT) and dihydrolipoamide S-succinyltransferase (DLST). GAPDH is the loading control.

E. Fractional enrichment of m+4 IMP, m+4 AMP, and m+4 GMP in WT and LIPT1-deficient (*LITP1^D*) cells during 6 hours of [$^{15}N_4$]hypoxanthine tracing (n=3).

F. Western blot validating deletion of HPRT1. Vinculin is the loading control.

G. Relative HPRT1 enzymatic activity in lysates from H460 cells treated with DMSO or 25 nM IACS-010759 for 24 hours (n=3).

H. Relative abundance of R5P in H460 cells treated with DMSO or 25 nM IACS-010759 for 24 hours (n=3).

I. Schematic illustrating R5P labeling from [U-^{13}C]glucose.

J. Relative abundance of R5P during [U-^{13}C]glucose tracing in H460 cells pre-treated with DMSO or 25 nM IACS-010759 for 24 hours (n=3).

K. Time-dependent m+5 enrichment in R5P from [U-^{13}C]glucose in H460 cells pre-treated with DMSO or 25 nM IACS-010759 for 24 hours (n=3).

L. Time-dependent fractional enrichment of m+6 6-PG and m+7 S7P during [U-^{13}C]glucose tracing in H460 cells pre-treated with DMSO or 25 nM IACS-010759 for 24 hours (n=3).

Unpaired, two-sided t tests (**A, B, E, G** and **H**) and two-way ANOVA test (**C,** and **J-L**) were used for the statistical analyses. ****: $P < 0.0001$; ***: $P < 0.001$, **: $P < 0.01$, *: $P < 0.05$; n.s.: $P > 0.05$. Error bars denote SEM. BioRender was used to generate the illustration.

Supplemental Figure 6 (Related to Figure 6)

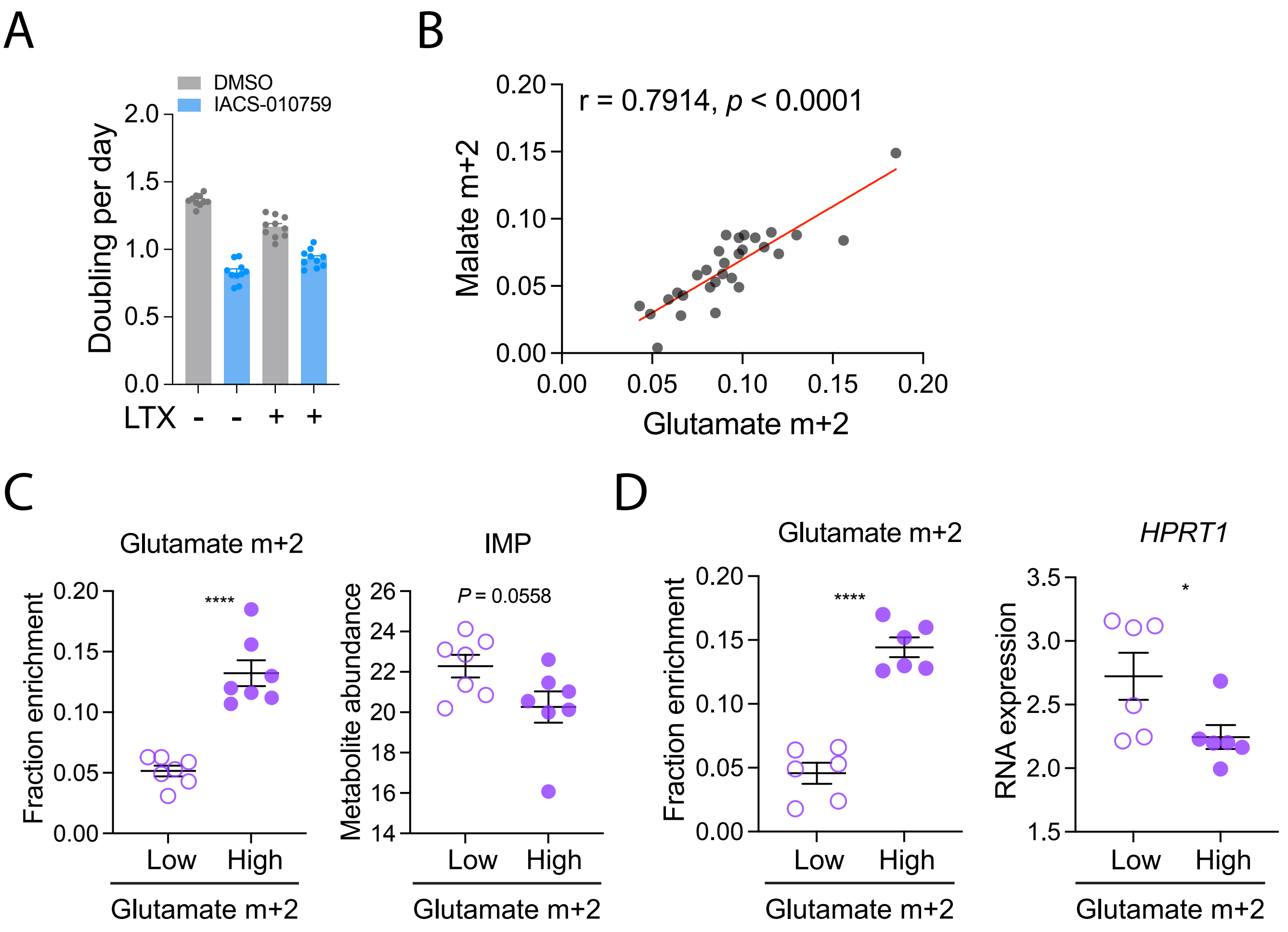

Figure S6. Analysis of purine and mitochondrial oxidative metabolism in human NSCLC in vivo, related to Figure 6.

A. Growth rates of H460 cells treated with DMSO, 25 nM IACS-010759, 5 µM LTX, or both IACS-010759 and LTX. Data are from one of three independent experiments.

B. Correlation between m+2 malate and m+2 glutamate in human NSCLC tumors subjected to intra-operative infusion with [U-^{13}C]glucose.

C. Fractional enrichment of m+2 glutamate and relative IMP abundance in human NSCLCs with high or low glutamate labeling. The analysis used tumors in the top or bottom 25% of glutamate m+2 labeling (n=7 of each).

D. Fractional enrichment of m+2 glutamate and *HPRT1* mRNA levels in human NSCLCs with high or low glutamate labeling. The analysis used tumors in the top or bottom 25% of glutamate m+2 labeling (n=6 of each).

Supplemental Figure 7(Related to Figure 7)

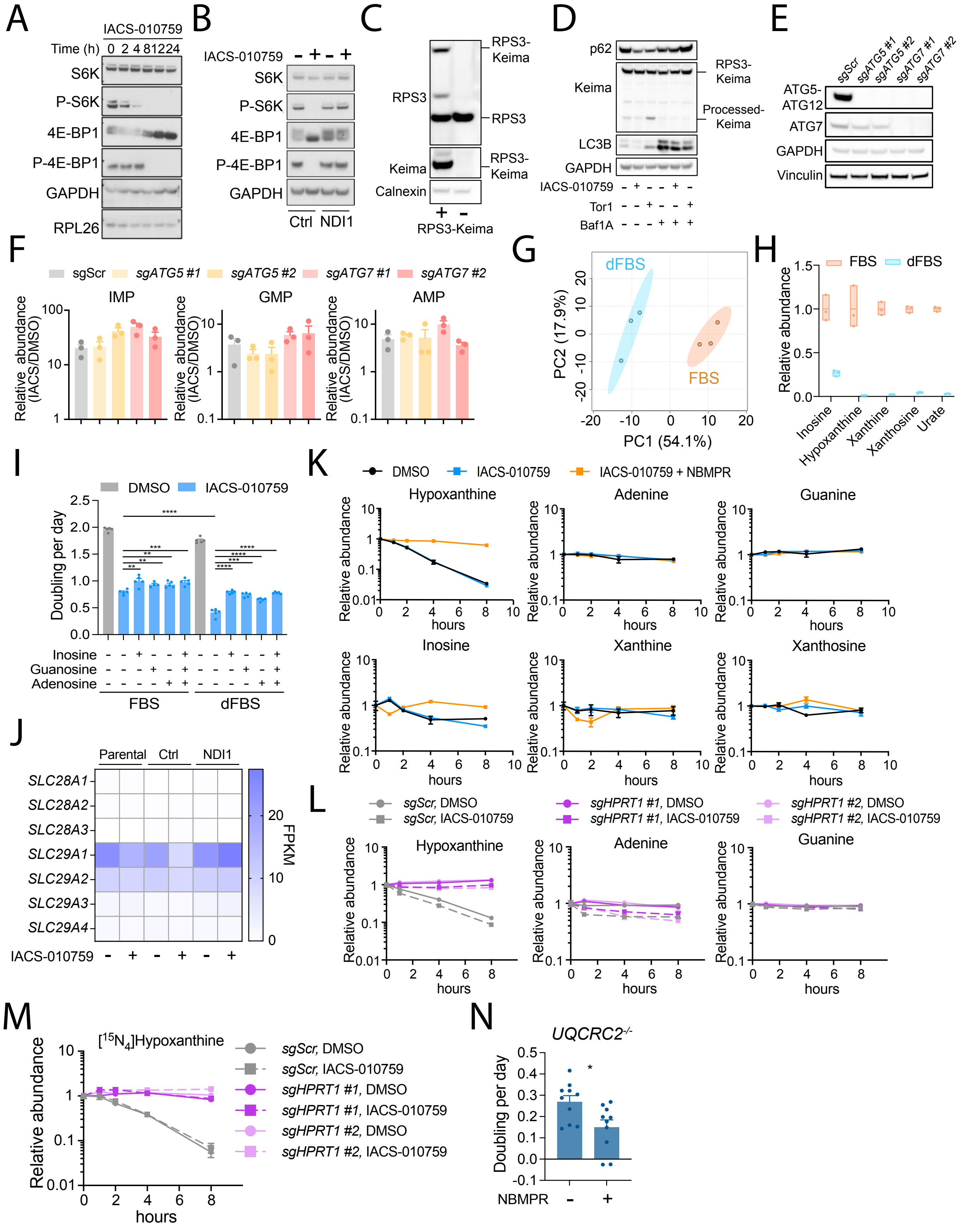

Figure S7. ETC-deficient cells utilize extracellular purine nucleosides and bases for purine salvage, related to Figure 7.

A. Western blot assessing mTORC1 signaling in H460 cells treated with 25 nM IACS-010759. GAPDH and RPL26 are loading controls.

B. Western blot assessing mTORC1 signaling in control and NDI1-expressing H460 cells treated with DMSO or IACS-010759 for 24 hours. GAPDH is the loading control.

C. Western blot validating RPS3-Keima expression in H460 cells. Calnexin is the loading control.

D. Western blot assessing ribophagy and overall macroautophagy in RPS3-Keima-expressing H460 cells treated with DMSO or the indicated compound(s) for 24 hours. GAPDH is the loading control.

E. Western blot validating ATG5 and ATG7 deletion. Vinculin and GAPDH are loading controls.

F. Relative abundance of the indicated purine nucleotides after 24 hours of DMSO or 25 nM IACS-010759 treatment in control (sgScr), ATG5-deficient (sgATG5), and ATG7-deficient (sgATG7) H460 cells (n=3).

G. Principal component analysis of metabolomic profiles in FBS and dialyzed FBS (dFBS).

H. Relative abundance of the indicated purine metabolites in FBS and dialyzed FBS (dFBS) (n=3).

I. Growth rates of H460 cells treated with 25 nM IACS-010759 and cultured in medium supplemented with FBS or dialyzed FBS (dFBS) and containing or lacking a mixture of 50 µM adenosine, inosine, and guanosine nucleosides (n=10). Data are from one of three independent experiments.

J. Heatmap showing RNA levels of *SLC28A* and *SLC29A* family transporters in parental, control, and NDI1-expressing H460 cells with or without 24 hours of IACS-010759 treatment.

K. Relative extracellular abundance of the indicated purine metabolites over 8 hours of culture of H460 cells treated with DMSO, 25 nM IACS-010759, or both 25 nM IACS-010759 and 50 µM NBMPR (n=3 at each time point).

L. Relative extracellular abundance of the indicated purine metabolites over 8 hours of culture of (*sgScr*) or HPRT1-depleted (*sgHPRT1*) H460 cells treated with DMSO or 25 nM IACS-010759 (n=3 at each time point).

M. Relative extracellular abundance of [$^{15}N_4$]hypoxanthine over 8 hours of culture of control (*sgScr*) or HPRT1-depleted (*sgHPRT1*) H460 cells treated with DMSO or 25 nM IACS-010759 (n=3 at each time point).

N. Growth rates of *UQCRC2*$^{-/-}$ cells treated with DMSO or 50 µM NBMPR (n=10). Data are from one of three independent experiments.

Unpaired, two-sided t test were used for the statistical analyses. ****: P < 0.0001; ***: P < 0.001; **: P < 0.01. Error bars denote SEM.
